# Correlated structure of neuronal firing in macaque visual cortex limits information for binocular depth discrimination

**DOI:** 10.1152/jn.00667.2020

**Published:** 2021-05-12

**Authors:** Jackson E. T. Smith, Andrew J. Parker

**Affiliations:** Department of Physiology, Anatomy and Genetics, University of Oxford, Oxford, United Kingdom

**Keywords:** behavior, cortex, noise correlation, rhesus, stereo depth

## Abstract

Variability in cortical neural activity potentially limits sensory discriminations. Theoretical work shows that information required to discriminate two similar stimuli is limited by the correlation structure of cortical variability. We investigated these information-limiting correlations by recording simultaneously from visual cortical areas primary visual cortex (V1) and extrastriate area V4 in macaque monkeys performing a binocular, stereo depth discrimination task. Within both areas, noise correlations on a rapid temporal scale (20–30 ms) were stronger for neuron pairs with similar selectivity for binocular depth, meaning that these correlations potentially limit information for making the discrimination. Between-area correlations (V1 to V4) were different, being weaker for neuron pairs with similar tuning and having a slower temporal scale (100+ ms). Fluctuations in these information-limiting correlations just prior to the detection event were associated with changes in behavioral accuracy. Although these correlations limit the recovery of information about sensory targets, their impact may be curtailed by integrative processing of signals across multiple brain areas.

**NEW & NOTEWORTHY** Correlated noise reduces the stimulus information in visual cortical neurons during experimental performance of binocular depth discriminations. The temporal scale of these correlations is important. Rapid (20–30 ms) correlations reduce information within and between areas V1 and V4, whereas slow (>100 ms) correlations between areas do not. Separate cortical areas appear to act together to maintain signal fidelity. Rapid correlations reduce the neuronal signal difference between stimuli and adversely affect perceptual discrimination.

## Graphical abstract

**Figure:**
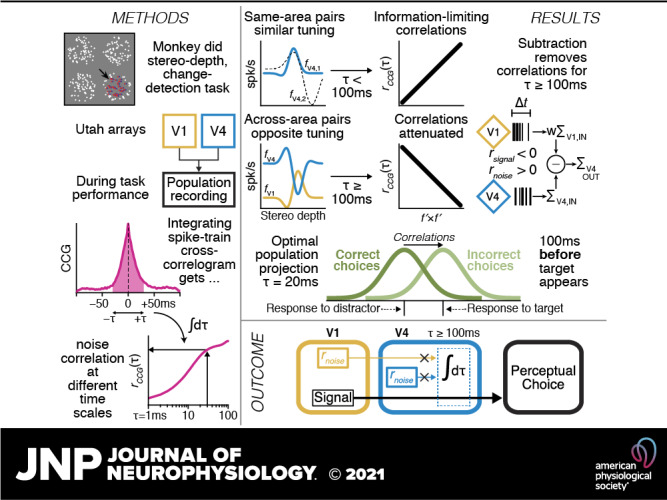

## INTRODUCTION

Processing in the sensory areas of cerebral cortex is thought to limit the detection and discrimination of weak sensory signals (1). Initial work examined how the changes in the firing of action potentials induced by near-threshold changes in the sensory stimulus could be detected against the variability of the base level of cortical activity (2–5). This work elucidated how detection and discrimination could be related to the statistics of the firing of single neurons.

These initial studies indicated that it was not unusual for the detection performance of single neurons to match the behavioral performance of the entire visual system. However, since the firing of all neurons shows some variability, combination of the firing from a pool of neurons to form a multineuron sensory signal ought to improve the detection rate for weak stimuli. The puzzle is why the nervous system apparently cannot provide a better sensitivity for the organism by making a statistical combination of the signals from multiple neurons.

One answer from early work on the somatosensory periphery is that the correlation of residual noise within a pool of sensory neurons may also be an important factor (6). In the absence of correlations, additive pooling of neuronal signals results in a steady improvement of sensory thresholds with increasing pool size. However, the presence of correlations limits the potential gain in performance from increases in the numbers of neurons. As more and more neurons are added, the portion of noise that is uncorrelated does indeed get averaged away, but the portion of noise that is correlated, and therefore shared by every neuron, cannot be reduced this way and comes to dominate the limit on performance (1, 7).

### Correlation Structure in the Pool of Sensory Neurons

Recent work expands that insight, showing how the nature of the pooling computation and the structure of correlations are both critical in setting limits on the encoding and decoding of information within sensory cortex (8–12). The correlation structure of a pool of neurons in relation to sensory discriminations is captured by making all pairwise comparisons between members of the pool across a number of repeated presentations of the sensory stimuli. Critically for our study, this theoretical work demonstrates that the signals from a neuron pair limit the discrimination between two stimuli in two ways (13). The first is fairly straightforward, which is that the change in the stimulus should alter the mean level of firing from both neurons; bigger differences in the mean response should improve the discrimination ability. The second concerns the variability of neuronal firing and its correlation structure across the population. The theoretical work demonstrates that noise or variability is the most detrimental when its correlated structure causes the conjoint firing of the neuron pair to mimic a response to some change in the stimulus that did not, in fact, occur. Such correlations have been termed “information-limiting correlations” (11, 14).

Assessment of the correlation structure ideally requires the simultaneous recording of multiple pairs of neurons from within the sensory cortex during the performance of a behavioral task with repeated stimulus presentations (11, 12). In our work, we addressed this by training monkeys to discriminate binocular stereoscopic depth while we tested a range of neuronal encoding by using chronically implanted Utah arrays to record multiple neurons simultaneously in two cortical sites: the primary visual cortex (V1) and the extrastriate area V4. Although the fact that we studied binocular vision is not especially critical for the questions that we investigate here, the processing of stereoscopic information proves to be a good model system for isolating the contribution of cortical processing, as stereo involves the direct combination of information from the two eyes at a cortical level.

### Neuronal Recording during Sensory Task Performance

Our task was designed so that we could thoroughly test all simultaneously recorded neurons over a range of stereoscopic depths, to build up a complete tuning curve for each neuron. Many studies have employed tasks that involve a binary decision between just two stimulus classes, for example, upward and downward motion. This strategy potentially limits which cortical neurons are relevant to the task, because many neurons are unresponsive to the difference between the two stimulus classes. Effective encoding of sensory stimuli by a neuron takes place only when the neuron’s activity is differentially altered by different stimuli. For single-unit recording studies, it is generally possible to adapt the stimulus parameters, such as motion direction, for each recorded neuron to ensure a strong modulation of neuronal firing along the sensory decision axis (5).

When recording multiple single neurons, it is harder to fulfill this goal simultaneously for all available neurons. As a result, during a simple binary decision task many of the recorded neurons may never be tested under conditions that probe their ability to encode sensory information. For this reason, it is better to design the experiment and behavioral task so that a wider range of neuronal selectivity and stimulus preferences can be tested. For our experiments here, we arranged for the same behavioral judgment to be performed across a range of different baseline values for stereoscopic depth, thereby increasing the opportunity to measure the neuronal responses around the point of greatest modulation by stereoscopic depth. In the case of using fixed recording electrodes, such as the Utah array, measurement over the wide range of sensory encoding also increases the yield of relevant neuronal responses.

The paradigm used here allowed us to estimate the extent to which pairs of neurons respond in the same way across a range of stimulus values, which is termed the signal correlation for the neuron pair (15). As our task involved the discrimination between two closely similar binocular depths, we also assessed the differential response of the sensory neurons by taking the slope of the tuning curve at each point. By making repeated measures of the response to each binocular depth, we could also estimate the noise correlation of all recorded neuron pairs at each tested value of binocular depth and apply recently developed methods to search for information-limiting correlations (12). We couple this approach with a time-dependent measure of correlations (15) to estimate the temporal scale of information encoding.

In earlier comparisons of the correlation structure and sensory tuning of cortical neurons, the data were either from anesthetized animals (16) or from awake animals that fixated without performing any other task (17). In other cases, the behavioral measures were taken on one task (judgment of heading direction), but the measures of correlation structure were taken during the performance of a different task (visual fixation) (18). In an alternative scenario, the measurements of signal correlation were obtained while the animal was passively fixating rather than actively discriminating the stimuli (19).

An important advance made with the recordings undertaken here is that the macaque monkeys actively performed a behavioral task, always making a stereoscopic depth judgment on the presented stimuli. We obtained simultaneous neuronal and behavioral data across the complete tuning curve of the neurons. A number of recent studies have demonstrated that the correlation structure of neuronal firing in sensory areas is influenced by the manipulation of cognitive factors such as attention or perceptual learning (19–23). We aimed to maintain these factors as constant as possible to focus on the discrimination performance of the neuronal population, by ensuring that the design of the experimental task arranged for spatial and featural attention to be consistently directed toward the depth signaled by the visual stimuli.

Finally, the relationship between perceptual decisions and the correlation structure of neuronal firing has been a topic of intensive research (18, 24–26) ever since it was suggested that the two may be linked (7). To assess this link, it is critical to measure the correlation structure in the most relevant neuronal population as the animal is performing a sensory discrimination task. To perform a discrimination task effectively requires that there is a statistically significant change in neuronal activity when comparing the responses to the two stimuli that must be discriminated. In respect of a conventional tuning curve for sensory stimuli, this means the points on the tuning curve where the slope of tuning curve is greatest, giving the biggest differential response between two neighboring stimuli. In this article, we are primarily concerned with the state of cortical firing as the animal waits attentively for the arrival of a change in binocular depth that must be detected and reported. Our results show for the first time that the state of putative differential correlations within the neuronal population has a direct association with the accuracy of the upcoming behavioral decision.

### Correlations within and between Cortical Areas

A further open question is whether the noise correlation structure limits the passage of signals through each of the stages of cortical processing (27). We tackle this issue by examining the correlation structure and information encoding of neuronal activity within and between cortical areas V1 and V4. Specifically, we test how information may pass between cortical areas, with respect to neuronal correlations during task performance. We examine the time course of information-bearing signals and assess them in relation to information-limiting correlations and behavioral choice. Our results suggest a simple model, which demonstrates that the potentially deleterious effects of information-limiting correlations do not build up as sensory signals pass from one cortical area to another. We suggest that this is an important and previously unrecognized principle of neural encoding across multiple areas of the cerebral neocortex.

## METHODS

### Animals

We recorded from two adult male rhesus macaque monkeys (*Macacca mulatta*) (*M135* and *M138*) acquired from the UK Public Health England breeding colony at Porton Down through the Center for Macaques. Each animal was implanted with a titanium headpost (Gray Matter Research, Bozeman, MT) and a CerePort pedestal (Blackrock Microsystems, Salt Lake City, UT) in separate aseptic surgical procedures under general anesthesia. Each CerePort was connected to a pair of 64-channel Utah arrays that were implanted subdurally in the upper layers of the exposed cerebral cortex. The exact locations for surgical implantation were determined by a preliminary magnetic resonance imaging (MRI) scan of the animal’s head to reveal bone and soft tissue structures.

Animals were trained to fixate on a target and then to perform an odd-one-out task using saccadic eye movement responses. All procedures were performed in accordance with United Kingdom Home Office regulations and European Union guidelines on animal experimentation (EU Directive 2010/63/EU). Animal protocols passed local and national ethical review and were licensed by the UK Home Office (Project licence Numbers P90964273 and 30/3041). The animals were given free access to food and water every day. The key results were qualitatively similar in both animals, and so we have pooled the data across animals, unless otherwise stated.

### Visual Stimulus

Visual stimuli were presented with a Wheatstone stereoscope comprising a pair of CRT monitors of the same model/manufacturer (Flexscan F78, Eizo, Hakusan, Japan; or P225f, ViewSonic, Brea, CA) at 84 cm from the subject’s eyes. Cold mirrors were used to reflect images into the eyes so that the infrared light from an eye tracking camera (Hi Speed Primate, SensorMotoric Instruments GmbH, Teltow, Germany), set behind the mirrors, could pass through. Monitors had a resolution of 1,600 × 1,200 pixels and a refresh rate of 85 Hz and subtended 26.7° × 20.1° of the subject’s visual field. Each pixel was 1.6e−2° wide. The VGA signal was split and duplicated, so that the left eye monitor received three copies of the red channel for its RGB input while the right eye monitor received three copies of the green channel. When viewed on a conventional single-color monitor by human observers the stereo images were visible as red/green anaglyph images, but viewed through the stereoscope the images appeared as grayscale images presented to each eye of the subjects. Thus, the Wheatstone stereoscope produced no stereoscopic cross talk.

Quadro K2200 (NVIDIA, Santa Clara, CA) video cards in multicore Intel (Santa Clara, CA) processor computers running Ubuntu 14.04 (Linux kernel 4.4.0 lowlatency) were used to drive the monitors. Stimuli were programmed with PsychToolbox (3.0.14; Ref. 28) running in MATLAB R2015b (The MathWorks, Natick, MA). All stimuli were presented on a midgray background, using appropriate OpenGL alpha-blending (GL_SRC_ALPHA + GL_ONE_MINUS_SRC_ALPHA) to obtain subpixel resolution. A 4 × 4-cm square was shown in the upper left corner of each monitor during trials. This was recorded with a photodiode (PIN-25DP, OSI Optoelectronics, Hawthorne, CA) by the electrophysiology system, in order to synchronize high-precision frame time stamps from PsychToolbox with the neural activity. The square was blocked from the subject’s view. A full white 0.3°-diameter circular dot was used as the gaze fixation target.

Random dot stereograms (RDSs) were used to present binocular disparities for the behavioral, odd-one-out task and to stimulate recorded neurons. Each RDS comprised a circular patch of 0.16°-diameter circular dots. Dot positions were randomly sampled on each frame; the frame rate matched the refresh rate of 85 Hz. Four RDSs were presented, but dot positions were sampled independently for each RDS. Half of the dots were black, and half were white. Dots occluded each other in a random order. Each RDS had two regions, a 6°-diameter circular center and a 1°-wide annular surround. The dot density in both regions was 25% (area occupied by dots/total area), assuming no dot overlap; in other words, the number of dots per RDS was 25% of the RDS area divided by the area of a single dot, rounded up. The RDS center varied in binocular disparity such that a flat circular plane of dots was shifted convergently (negative disparities, toward subject) or divergently (positive, away). Surround dots were always on the fixation plane at zero disparity, i.e., the surface of the monitors’ screens. The surround functioned to mask monocular cues when nonzero disparities were presented in the center and to help the subject maintain vergence. When the RDS center was at a nonzero disparity, each of its monocular images had a horizontal shift that overlapped the RDS surround on one side while leaving a gap on the other. Surround dots in the overlap region were discarded, whereas uncorrelated dots were used to fill the gaps, maintaining a uniform dot density, on average, across the RDS. Thus, the resulting monocular images carried no trace of the disparity of the RDS center, so that the subjects could only perform the task using the stereoscopic information.

### Task

**Figure 1:**
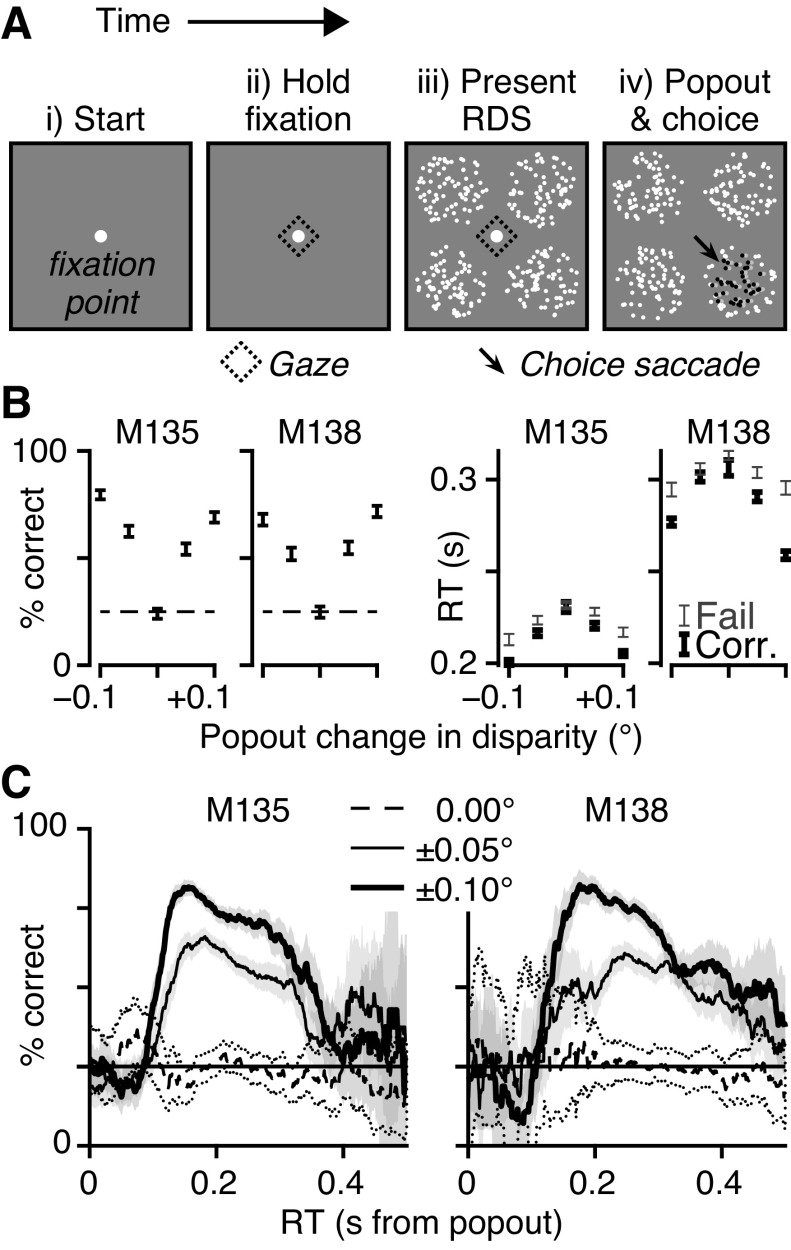
Task and performance. *A*, *i*: trials started with a flashing fixation point (FP, white circle). *ii*: Once fixated (dashed diamond), FP flash stops. *iii*: 500 ms later, 4 random dot stereograms (RDSs, white dots) with identical properties appeared. *iv*: After fixed duration (1 s *M135*, 2 s *M138*), central dots of 1 RDS change disparity (gray/black dots) and FP disappears. Animals made saccade onto the oddball target RDS (arrow) for reward. *B*: % correct trials (chance = 25%, dashed line) and reaction time (RT) over popout disparity. Trials with 0.117 ≤ RT ≤ 0.344 s for *M135* (*N* = 6,670 of 8,279) or 0.143 ≤ RT ≤ 0.463 s for *M138* (*N* = 5,478 of 6,073). RT shown separately for correct (thick) and incorrect (thin) choices, i.e., failed trials. Error bars, 95% binomial confidence intervals (CIs) or standard error of the mean (SE). *C*: % correct across RT using 50-ms moving window; with 95% bootstrap CI (2,000 samples, 2.5th and 97.5th percentiles). Data grouped by absolute popout disparity (see key).

Subjects performed an odd-one-out detection task (Fig. 1*A*) that was divided into four epochs. At the start of each trial, a fixation point was presented (Fig. 1*Ai*). The subject then had to maintain its gaze at the fixation point for 0.5 s to initiate the trial (Fig. 1*Aii*). after this, four RDSs were presented for 1 s (*M135*) or 2 s (*M138*), one in each quadrant of the visual field (Fig. 1*Aiii*). Subjects had to maintain their binocular gaze within a 0.75° radius of the center of the fixation dot to both initiate the trial and progress through to the end of the presentation phase. During the presentation phase, all four RDSs had the same baseline disparity in their central region. Baseline disparities were randomly selected on each trial with equal probability from a set of 13 values: 0°, ±0.01°, ±0.02°, ±0.05°, ±0.10°, ±0.20°, and ±0.50° (similar to Ref. 29). At the end of the presentation period, one of the four RDSs had a disparity step change in its central region, gaining an additional 0°, ±0.05°, or ±0.10° (Fig. 1*Aiv*). Both the location and value of the step change were randomly selected on each trial with equal probability. Thus, the RDS that changed became the odd one out (i.e., the popout target or oddball), which the subject was required to detect by making a saccade onto its location. With four possible target locations, the probability of correctly selecting the target by chance was 0.25 (Fig. 1*B*).

A juice reward was given for correct performance. No reward was given for incorrect saccades or if the subject responded before the step change. On trials with a step change of 0°, the subject was rewarded for guessing the notional target location. After the training or testing session, animals had ad libitum access to water in the home enclosure for a limited period of time every day. Animals were weighed each day and assessed.

A unique dot sequence was generated in each RDS for each trial. This is an essential requirement because it prevents the subject from ever learning that a specific dot sequence is attached to a particular disparity or target location. If the same sequence had been repeated across trials and days, then the subject could have access to an alternative, monocular cue. By presenting unique dot sequences, we forced the subjects to use only the binocular disparity cue.

### Recording

The animals’ binocular gaze positions were sampled at 500 Hz and recorded alongside the neural responses. In cortex, each subject was implanted with two Utah arrays (Blackrock Microsystems). Both arrays had 64 platinum electrodes, each 1 mm in length, arranged in an 8 × 8 grid with 400-μm spacing. It is unclear exactly which layer of the cortex each electrode tip had entered, because of the curvature of the cortical surface relative to the flat array and from slight deviations of the arrays from the plane tangential to the cortex. Nonetheless, given the electrode lengths, the presumption is that the recordings reported here are primarily from the upper, supragranular layers of the cortex. One array was implanted in cortical area V1 and the other in V4. A CerePlex E headstage (Blackrock Microsystems) was attached to the CerePort implant. The headstage applied 0.3-Hz first-order high-pass and 7.5-kHz third-order low-pass antialiasing Butterworth filters to the analog signals before 16-bit digitizing all 128 channels at 30 kHz. Digitized signals were transmitted to the Cerebus (128-channel electrophysiology system, Blackrock Microsystems) using fiber optics, thus minimizing noise at the analog stage. All computers, monitors, and the reward pump were located in a separate control room, whereas the subject, headstage, stimulus monitors, digital hub, and CRTV camera were within a test room that had Faraday shielding. A second-order 250- to 5,000-Hz band-pass digital Butterworth filter was applied online to each data channel before application of a threshold of −6.5 × the root mean square (RMS) of the estimated noise level to detect action potentials (30); the thresholds were newly estimated on each experiment for each electrode. The −6.5 × RMS threshold was the default setting for the Cerebus, which worked well in practice to detect multiunit activity without saturating the acquisition system and losing data.

The simultaneous V1/V4 data in this study came from six recording sessions (1 session/day) with *M135* and four sessions with *M138*, yielding 8,279 trials from *M135* [1,380 per-session mean (60.7 standard deviation)] and 6,073 from *M138* [1,518 (58.0)]. We made no assumptions about the identity of each unit, treating each day’s recording separately. Here, the term “unit” simply means a cluster of spike waveforms from one electrode in one recording session (see below). This yielded a total of 63 V1 [10.5 (3.2)] and 111 V4 [18.5 (3.5)] units from *M135* and 132 V1 [33.0 (3.5)] and 102 V4 [25.5 (3.7)] units from *M138*. Thus, we retrieved 315 V1 [52.5 (32.6)], 975 V4 [162.5 (63.5)], and 1,206 intercortical [201.0 (91.6)] neuron pairs from *M135* and 2,104 V1 [526.0 (108.4)], 1,251 V4 [312.8 (90.6)], and 3,392 intercortical [848.0 (183.7)] pairs from *M138*. All pairwise analyses compared units from separate electrodes (see Figs. 3–6 and 8).

When trials were grouped by baseline disparity, there was an average of 106 trials (SD = 6.2 trials) per group for *M135* and 116 trials (4.8 trials) for *M138*. Across experimental days, the smallest group size was 97 trials (4.8 trials) on average for *M135* and 111 trials (4.5 trials) for *M138*.

For the behavioral analysis in Figs. 9 and 10, trials were grouped on the basis of two factors. First, the target RDS could be IN or OUT of receptive field (RF). Second, the response to a trial could be CORRECT or INCORRECT. In addition, trials were only used if the reaction time (RT) was in the range 117 ms ≤ RT ≤ 344 ms for *M135*, and 143 ms ≤ RT ≤ 463 ms for *M138* (see Fig. 1*C*). When the target was IN the RF, then the average number of correct trials across the days of recording was 217 [standard deviation (SD) 14.6] correct and 60 (SD 14.4) incorrect trials for *M135* and 228 (SD 12.2) correct and 111 (SD 14.5) incorrect trials for *M138*. Alternatively, when the target was OUT of the RF then the daily averages were 432 (SD 33.2) correct and 237 (SD 32.4) incorrect trials for *M135* and 520 (SD 34.2) correct and 265 (SD 35.4) incorrect trials for *M138*.

**Figure 2.**
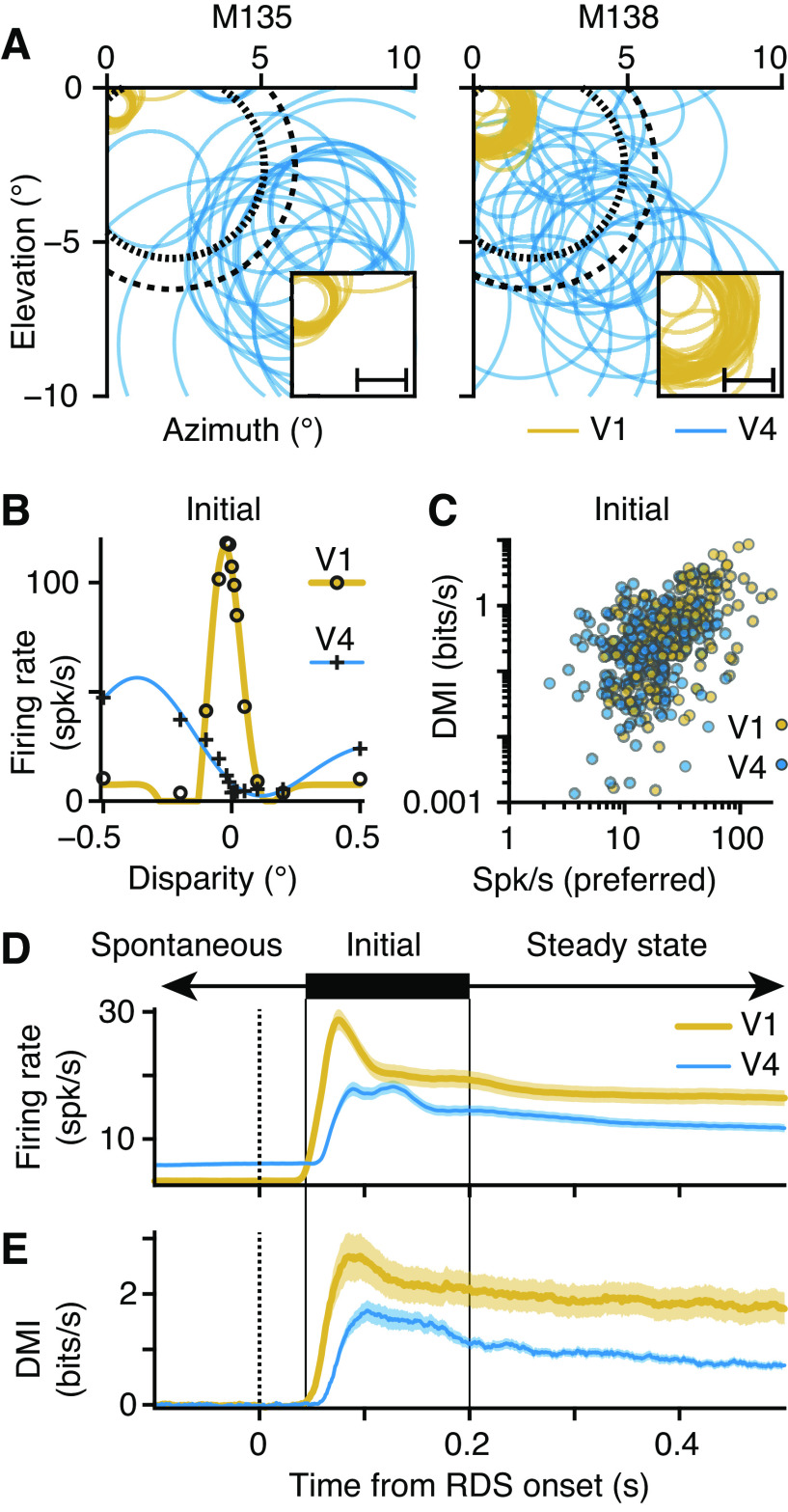
Primary visual cortex (V1) and extrastriate area V4 units were selective for binocular disparity. *A*: receptive field (RF) location and size for each V1 (gold) and V4 (blue) neuron; each circle is the contour line at 1 standard deviation on the 2-dimensional Gaussian fit (least squares) to the empirical RF location map. Random dot stereogram (RDS) center (short dash) and surround (long dash) are shown. *Insets*: magnified V1 RFs. Scale bars, 1°. *B*: example disparity tuning curves from V1 (circles, yellow) and V4 (crosses, blue); symbols show average firing rates; lines show best-fitting Gabors: SE below symbol size. *C*: disparity mutual information (DMI) for V1 (gold, *N* = 195) and V4 (blue, *N* = 213) units compared with average firing rate to preferred disparities. *D*: average firing rate (line, mean; shading, SE) across all V1 (gold) and V4 (blue) units, over trial sequence: analysis windows cover spontaneous responses (left arrow), Initial phasic response (solid bar and lines) and later Steady state responses (right arrow); dashed line marks the onset of RDS. *E*: as in *C* but for DMI. spk, Spikes.

To provide effective visual stimulation of both V1 and V4 receptive fields (RFs) with the central region of the lower right RDS, the array of all four RDS stimuli was sometimes shifted upward and to the left, in which case they had an asymmetric arrangement relative to the fixation point (Fig. 2*A*).

### Analysis

The data were analyzed with programs that were written and executed in MATLAB R2015b and R2019a.

#### Preprocessing.

In both animals, we found incidences of cross talk between a subset of electrodes. This was apparent as a fraction of tightly synchronous waveform times between certain pairs of electrodes, defined as waveforms occurring within ±1.3e−4 s, i.e., ±4 samples at 30 kHz of each other. Synchronous waveforms often had highly similar, stereotypical shapes. Testing with a digital neural signal simulator (Blackrock Microsystems) indicated that all components downstream of the CerePort functioned properly. Subsequent testing by the manufacturer suggests that there was a larger tolerance of fit between some pairs of CerePort and CerePlex E than had been specified, causing small amounts of misalignment on some recording days that could explain the emergence of cross talk on some groups of channels. Thus, individual electrodes in the Utah arrays were discarded from the analysis if they had at least 6% synchronous spikes with any other electrode and the median Pearson correlation of the synchronous waveforms was 0.5 or more. An electrode was also discarded from analysis if there was no significant change in the multiunit spiking rate after onset of the RDS stimulus. This was defined by counting spikes in two 500-ms-wide analysis windows, one from −500 ms to the appearance of the RDS and the other from 50 ms to 550 ms. A paired-sample *t* test was used to compare the firing rates before and after RDS appearance at a significance level of 5%. When a significant difference was found, then the electrode’s spikes were sorted for further analysis. On average, 9 V1 (minimum 5, maximum 14) and 14 V4 (min 12, max 17) electrodes from *M135* were used and 27 V1 (min 22, max 31) and 20 V4 (min 19, max 23) electrodes from *M138* were used per session.

Spikes detected online with the Cerebus were sorted with our implementation of the clustering algorithm by Fee et al. (31) and the heuristic choices of UltraMegaSort2000 (Ref. 32; https://neurophysics.ucsd.edu). An additional step was taken after aligning the spike waveform peaks but before the principal component analysis (PCA). Each waveform was weighted by a Gaussian-shaped window with a standard deviation of 1 ms that was centered on the waveform peak; area under the Gaussian that overlapped the waveform was normalized to preserve the overall magnitude of the spike. This emphasized the peak region of the waveforms during sorting. Automated spike sorting was then verified and corrected manually. We based our analyses on both single units and multiunit clusters and use the term “unit” to refer to either, as in other work with Utah array electrodes (21) (see also Ref. 33 for work with Neuropixels probes).

Disparity tuning curves for prescreening were found for each unit, using spike rates from an analysis window of 50 to 1,050 ms (*M135*) or 50 to 2,050 ms (*M138*) after onset of the RDS stimuli. Only units with significant disparity tuning (1-way ANOVA, *P* < 0.01) were kept for further analysis. Additionally, any unit with a tuning curve peak of <5 spikes/s was also discarded. These tuning curves were not used in the main analysis.

#### Single-unit analysis.

The selectivity of a unit for binocular disparity was quantified in two ways. Disparity discrimination index (DDI; Ref. 34) was used to validate disparity mutual information (DMI). DDI was defined as

(*1*) $$\frac{N_{\max}-N_{\min}}{\left(N_{\max}-N_{\min}\right)+2R_{\mathrm{error}}}$$

where *N*_max_ and *N*_min_ are the largest and smallest responses of the unit’s disparity tuning curve and *R*_error_ is the square root of the residual variance around the means over the whole tuning curve. DDI was computed with the square root of the firing rates (as in Ref. 34).

DMI was computed nonparametrically (35) by first estimating the joint probability distribution of the disparity and spike count using 13 evenly spaced bins in both marginal dimensions to get a two-dimensional histogram (1 marginal bin per disparity value). Disparities were ranked ordinally before binning. Joint probabilities were empirically estimated by

(*2*) $$P_{xy}\left(i,j\right)=\frac{1}{M}\overset{M}{\underset{m=1}{\sum }}J_{m}\left(i,j\right)$$

where *M* is the total number of trials and *J_m_*(*i*,*j*) has a binary value:

(*3*) $$J_{m}\left(i,j\right)=\{\begin{array}{c} 1,\;\mathrm{ordinal}\;\mathrm{disparity}\;\mathrm{was}\;\mathrm{in}\;\mathrm{bin}\;i\;\mathrm{and}\;\mathrm{spike}\;\\ \mathrm{count}\;\mathrm{was}\;\mathrm{in}\;\mathrm{bin}\;j\;\mathrm{on}\;\text{trial}\;m\\ 0,\;\mathrm{otherwise} \end{array}$$

The marginal probabilities were computed in a similar manner. For ordinal disparity values (*P_x_*) and spike counts (*P_y_*), they were

(*4*) $$P_{x}\left(i\right)=\frac{1}{M}\overset{M}{\underset{m=1}{\sum }}x_{m}\left(i\right)$$

(*5*) $$P_{y}\left(j\right)=\frac{1}{M}\overset{M}{\underset{m=1}{\sum }}y_{m}\left(j\right)$$

where *x_m_*(*i*) and *y_m_*(*j*) have a value of 1 if the data fell into the respective bin on trial *m* and 0 otherwise. The raw empirical mutual information was estimated by

(*6*) $$I_{\mathrm{raw}}=\overset{B}{\underset{i=1}{\sum }}\overset{B}{\underset{j=1}{\sum }}P_{xy}\left(i,j\right)\mathrm{lo}g_{2}\frac{P_{xy}\left(i,j\right)}{P_{x}\left(i\right)P_{y}\left(j\right)}$$

where *B* is the number of bins along the marginal. However, this information estimate will be biased—without further correction. The correction was found by applying *Eq. 6* to randomly shuffled copies of the data. Using 30 repeats (36), a shuffle-correction term was estimated as

(*7*) $$I_{\mathrm{shuffle}}=\frac{1}{30}\overset{30}{\underset{s=1}{\sum }}i_{\mathrm{shuffle}}\left(s\right)$$

where *i*_shuffle_(*s*) is the output of *Eq. 6* following the *s*th random shuffling of the data. The final, corrected mutual information between the disparity and spike count was taken as

(*8*) $$I=\frac{I_{\mathrm{raw}}-I_{\mathrm{shuffle}}}{\text{Δ}t}$$

where Δ*t* is the width of the spike-counting analysis window, in seconds, providing a value in bits/second.

A time series analysis of firing rates and DMI was conducted on spike trains that were convolved with a causal exponential kernel (20 ms time constant). DMI was measured independently for each time point by using Δ*t* = 0.02 s (*Eq. 8*) to estimate the instantaneous rate of information. The firing rate and DMI time series were used to define three analysis windows for the pairwise and population analyses. The Spontaneous window started 500 ms before RDS onset, ended 50 ms after RDS onset, and covered the period of gaze fixation before the units’ first responses to the RDS. The Initial window was located to cover the first transient response to the RDS, which was measured from 50 ms to 200 ms after the RDS appeared. A final Steady state window ran from 200 ms to 1,050 ms for *M135* and from 200 ms to 2,050 ms for *M138*, covering responses to the RDS up to but not including those to the disparity step change.

#### Paired signal and noise correlations.

In the analysis of pairs of neurons, we focused on three key metrics, as defined by Bair, Zohary, and Newsome (15). First was the degree to which the firing rate of two units was driven by the same stimulus values—the pair’s signal correlation. Signal correlation was defined as the Pearson correlation of the pair’s disparity tuning curve values:

(*9*) $$r_{\mathrm{signal}}=\frac{E\left[N_{j}^{d}N_{k}^{d}\right]-E\left[N_{j}^{d}\right]E\left[N_{k}^{d}\right]}{{\text{σ}}_{N_{j}^{d}}{\text{σ}}_{N_{k}^{d}}}$$

where $N_{j}^{d}$ and $N_{k}^{d}$ are the average spike counts of units *j* and *k* in response to a disparity *d*, *E* is the mean value of the average spike counts across disparity values, and σ is the standard deviation of the average spike counts across disparity values.

The second metric was the degree to which the residual variances in the tuning curves were shared by the two units—the pair’s noise correlation. If the stimulus value is fixed over a set of trials, then noise correlation can be quantified as spike count correlation (*r*_SC_) using

(*10*) $$r_{\mathrm{SC}}=\frac{E\text{[}n_{j}n_{k}\text{]}-E\text{[}n_{j}\text{]}E\text{[}n_{k}\text{]}}{{\text{σ}}_{n_{j}}{\text{σ}}_{n_{k}}}$$

where *n_j_* and *n_k_* are the spike counts on a single trial from units *j* and *k*, *E* is the mean value across trials, and σ is the standard deviation across trials. To pool across trials with different disparity values we first *z*-score normalized the spike counts of each unit for each subset of trials, grouped by disparity; *r*_SC_ was then computed as

(*11*) $$r_{\mathrm{SC}}=E\text{[}z_{j}z_{k}\text{]}$$

where *z_j_* and *z_k_* are the *z*-scored firing rates on a single trial for units *j* and *k*.

A limitation of *r*_SC_ is that it only measures noise correlation at the timescale of the spike-counting window, which is typically on the order of seconds. However, noise correlations may occur at a range of timescales, from near synchrony at the scale of milliseconds to slower modulations over seconds. Noise correlation can be estimated at different timescales in the same analysis window (Spontaneous, Initial, or Steady state) using *r*_CCG_, which is the normalized integral of the cross correlation between paired spike trains. By integrating from 0 to ±Δ*t* lags under the cross correlation, *r*_CCG_ measures rapid noise correlations when Δ*t* is small and includes slower noise correlations when Δ*t* is large. *r*_CCG_ converges upon *r*_SC_ when Δ*t* approaches the width of the analysis window. To compute *r*_CCG_, the spike train of unit *k* on trial *m* was first binned into a series of millisecond-wide bins; the spike train become a binary series of zeros and ones such that

(*12*) $$x_{k,m}\left(t\right)=\{\begin{array}{c} 1\text{, if unit }k\text{ fired an action potential during ms }t\text{ on trial }m\\ 0\text{, otherwise} \end{array}$$

If there were *M* trials and the analysis window was *T* ms long, then 1 ≤ *m *≤* M* and 1 ≤ *t *≤* T*. The peristimulus spike time histogram (PSTH) of unit *k* during time *t* was

(*13*) $$P_{k}\left(t\right)=\frac{1}{M}\overset{M}{\underset{m=1}{\sum }}x_{k,m}\left(t\right)$$

The spike train correlation function at τ ms of lag is

(*14*) $${\text{C}}_{jk}\left(\text{τ}\right)=\frac{1}{M}\overset{M}{\underset{m=1}{\sum }}\overset{T}{\underset{t=1}{\sum }}x_{j,m}\left(t\right)x_{k,m}\left(t+\text{τ}\right)$$

If *j *=* k*, then *C_jk_*(τ) is the auto-correlation; otherwise, if *j *≠* k*, then it is the cross correlation between units *j* and *k*. Likewise, the auto- or cross correlation of the PSTH was

(*15*) $$S_{jk}\left(\text{τ}\right)=\overset{T}{\underset{t=1}{\sum }}P_{j}\left(t\right)P_{k}\left(t+\text{τ}\right)$$

In fact, *S_jk_*(τ) serves as a shift predictor for *C_jk_*(τ). Thus, the integral of the shift-corrected spike train auto- or cross correlation was

(*16*) $$A_{jk}\left(\text{θ}\right)=\overset{+\text{θ}}{\underset{\text{τ}=-\text{θ}}{\sum }}\left[C_{jk}\left(\text{τ}\right)-S_{jk}\left(\text{τ}\right)\right]$$

where the area *A_jk_*(θ) was summed symmetrically from − θ to + θ ms of lag.

After this, *r*_CCG_ was defined as

(*17*) $$r_{\mathrm{CCG}}\left(\text{τ}\right)=\frac{A_{jk}\left(\text{τ}\right)}{\sqrt{A_{jj}\left(\text{τ}\right)A_{kk}\left(\text{τ}\right)}}$$

Using *Eq. 17*, *Eq. 10* for *r*_SC_ can be recast as

(*18*) $$r_{\mathrm{SC}}=\frac{A_{jk}\left(T\right)}{\sqrt{A_{jj}\left(T\right)A_{kk}\left(T\right)}}$$

where 1 ≤ τ ≤ *T*. When τ is small, then only noise correlations on a short timescale are measured, whereas when τ is large, slower noise correlations are estimated. We found *r*_CCG_ separately for each disparity value. The mean *r*_CCG_ across disparities was found by taking the weighted average, weighting by the number of trials per disparity condition.

A third metric of paired activity is the normalized spike train cross correlogram (CCG), which shows the relative timing of correlated spikes between the pair of units, defined here as

(*19*) $$\mathrm{CC}G_{jk}\left(\text{τ}\right)=\frac{C_{jk}\left(\text{τ}\right)}{\text{Θ}\left(\text{τ}\right)\sqrt{{\text{λ}}_{j}{\text{λ}}_{k}}}$$

(*20*) Θ(τ)=T−|τ|

where λ*_k_* is the average firing rate (spikes per second) of unit *k* and −*T* ≤ τ ≤ +*T*. CCG was also found separately for each disparity value, and the mean across disparities was weighted by the number of trials. For presentation purposes, CCGs were convolved with the symmetric 5-ms kernel [0.05, 0.25, 0.40, 0.25, 0.05] (37).

#### First derivatives of the tuning curves.

To estimate the first derivative of the disparity tuning curves, we found the best-fitting one-dimensional Gabor function for the tuning curves of each unit, in both the Initial and Steady state analysis windows (34, 38). The Gabor was expressed as

(*21*) $$G\left(d\right)=c_{0}+c_{1}e^{-\left(\frac{{\left(d-c_{2}\right)}^{2}}{2{\left(c_{3}\right)}^{2}}\right)}\cos\left(2\text{π}c_{4}\left(d-c_{2}\right)+c_{5}\right)$$

where *G*(*d*) is the value of the Gabor function at a disparity value of *d* and *c_i_* are the parameters that were optimized for each tuning curve. The baseline offset (*c*_0_) was constrained between zero and the maximum observed response on any trial. The amplitude of the Gaussian envelope (*c*_1_) was constrained between zero and twice the difference between the maximum observed response on any trial and the minimum. The horizontal offset of the Gaussian envelope (*c*_2_) was constrained between the minimum and maximum disparity values that were tested. The width of the Gaussian envelope (*c*_3_) was constrained between 0.1 and the total range of tested disparity values. The disparity frequency of the cosine (*c*_4_) was estimated from the power spectrum of the tuning curve by first linearly interpolating (function *interp1*, MATLAB) the *z*-scored tuning curve from the minimum to maximum tested disparity, at 0.001° steps. The discrete Fourier transform at frequency *f* [*y*(*f*); function *fft*, MATLAB] was estimated from the interpolated tuning curve, and the power spectrum at *f* was computed using

(*22*) $$p\left(f\right)=\frac{{\left|y\left(f\right)\right|}^{2}}{D}$$

where *D* is the number of interpolated points. We found the frequency between 0 and 50 cycles per degree that maximized the power spectrum (*f*_max_) and then constrained the disparity frequency to be *c*_4_ = *f*_max_ ± 0.1*f*_max_. The phase of the cosine (*c*_5_) was constrained to be within ±π. The parameters were fit by using the trust-region-reflective algorithm to minimize the square of the residual error (function *lsqcurvefit*, MATLAB). After optimizing the parameters for each tuning curve, we estimated the first derivative of the tuning curves at each tested disparity with

(*23*) $$\text{G}\prime =\frac{\text{G}\left(d+\text{Δ}d\right)-\text{G}\left(d-\text{Δ}d\right)}{2\text{Δ}d}$$

using a value of 0.5e−3° for Δ*d*. The Gabors better captured the responses to smaller disparities near zero, hence the nine smallest disparities were used to estimate *f*′ *× f*′.

The results in Fig. 6 were verified by using a second method to estimate the first derivative of the tuning curves, in which the empirical tuning curves were linearly interpolated at *d* ± Δ*d* and the difference was divided by 2Δ*d*, for each tested disparity value of *d*.

#### Population response analysis.

To estimate the ability of simultaneous neuronal responses to distinguish one disparity value from another, we calculated the bias-corrected linear Fisher information as defined by Kanitscheider, Coen-Cagli, Kohn, and Pouget (12); their code formed the basis of our own MATLAB implementation (39). Linear Fisher information quantifies the ability of a linear decoder to distinguish two sets of simultaneous population spike counts as they arise in response to two different stimulus values. Hence, this was calculated by

(*24*) $${\hat{I}}_{bc}\left(\text{τ}\right)={\frac{\text{Δ}N}{\text{Δ}d}}^{tr}\text{Σ}{\left(\text{τ}\right)}^{-1}\frac{\text{Δ}N}{\text{Δ}d}\left(\frac{2M_{\text{min}}-U-3}{2M_{\text{min}}-2}\right)-\frac{2U}{M_{\text{min}}\text{Δ}d^{2}}\;$$

(*25*) $$\text{Δ}d=d_{a}-d_{b}$$

(*26*) $$\text{Δ}N=N^{d_{a}}-N^{d_{b}}$$

(*27*) $$\text{Σ}\left(\text{τ}\right)=\left({\text{Σ}}^{d_{a}}\left(\text{τ}\right)+{\text{Σ}}^{d_{b}}\left(\text{τ}\right)\right)/2$$

where *d_a_* and *d_b_* are two of the tested disparity values (in degrees) such that *d_a_* ≠ *d_b_*, *N^d^* is a column vector of the average spike counts (in spikes) for *U* units in response to disparity value *d*, Δ*N*/Δ*d* is also a column vector in which each element of the column vector Δ*N* was divided by Δ*d*, *tr* is the transposition operator that turned Δ*N*/Δ*d* into a row vector, Σ*^d^*(τ) is the *U *×* U* covariance matrix (in spikes^2^) for disparity value *d* at a timescale of τ ms, *X*^−1^ is the inverse of matrix *X* (Moore–Penrose psudoinverse, function *pinv*, MATLAB) in spikes^−2^, and *Î_bc_*(τ), bias-corrected Fisher information, is a scalar value with a unit of degrees^−2^.

We randomly discarded trials so that each disparity value had the same number of trials as that which had the minimum (*M*_min_), as assumed in the derivation by Kanitscheider, Coen-Cagli, Kohn, and Pouget (12). In practice, few trials were discarded, with the remainder being well above the minimum required for the covariance matrix to be invertible, which is (*U *+* *2)/2. For units *j* and *k*, we defined elements of Σ*^d^*(τ) to be

(*28*) $${\text{Σ}}_{j,k}^{d}\left(\text{τ}\right)=r_{\mathrm{CCG}}^{d}\left(\text{τ}\right){\text{σ}}_{j}^{d}{\text{σ}}_{k}^{d}$$

where $r_{\mathrm{CCG}}^{d}\left(\text{τ}\right)$, ${\text{σ}}_{j}^{d}$ and ${\text{σ}}_{k}^{d}$ are respectively the *r*_CCG_ and spike-count standard deviations of units *j* and *k* on trials with disparity *d*. Thus, we could estimate the effect of noise correlations at different timescales on the linear Fisher information of the population response.

A theoretical maximum of linear Fisher information is obtained when all units in the population are decorrelated with each other such that the variance of their spike counts is statistically independent. This is often achieved numerically by shuffling the data. However, it is possible to estimate this value analytically without shuffling—using only the diagonal of the covariance matrix (called *I_bc,shuffle_* in Ref. 12):

(*29*) $${\hat{I}}_{bc,\mathrm{decorr}}={\hat{I}}_{bc,\mathrm{decorr}}\left(\text{τ}\right)=\frac{2U}{M_{\min}\text{Δ}d^{2}}\overset{U}{\underset{i=1}{\sum }}\frac{{\left(\text{Δ}N_{i}/\text{Δ}d\right)}^{2}}{{\text{Σ}}_{i,i}\left(\text{τ}\right)}-\frac{M_{\min}-2}{M_{\min}-1}$$

*Equation 29* emphasizes the fact that with this calculation the decorrelated linear Fisher information is the same for all timescales of noise correlation, because the auto-correlated value of *r*_CCG_ is always 1 (see *Eq. 17* when *j *=* k*) and ${\text{σ}}_{j}^{d}$ does not vary with τ (*Eqs. 27* and *28*).

To see whether linear Fisher information saturated with increasing population size, we first ordered a given set of units by the DMI of their Initial responses, from largest to smallest. Units were added to the neuronal pool one at a time in this order, and the Fisher information was computed at each step; that is, the most disparity-selective unit was added first, and the least selective unit was added last. The result was an information curve that was a function of pool size. Initial DMI was always used to order the units because their Initial responses were the most selective for disparity (see Fig. 2). This ordering imposed a saturating shape on even the decorrelated information curves. However, we could then examine whether the saturation worsened as a result of noise correlations. Information curves were found for the Initial and Steady state responses at timescales of 20 and 150 ms (Initial) or 20 and 800 ms (Steady state) and for decorrelated units (both). To average across experimental sessions and combinations of disparity values, it was necessary to normalize the information values in a way that would preserve the relative shape of information curves at different timescales. To do this, the curves were first grouped by experiment and disparity values. Normalization was then achieved within a group by

(*30*) $$I_{\mathrm{norm}}\left(\text{τ},s_{\mathrm{pop}}\right)=\frac{I_{\mathrm{raw}}\left(\text{τ},s_{\mathrm{pop}}\right)-{\text{β}}_{\mathrm{grp}}}{{\text{α}}_{\mathrm{grp}}}$$

where *I*_raw_(τ,*s*_pop_) is the value of *Î_bc_*(τ) or *Î_bc,_*_decorr_ at population size *s*_pop_ units. β_grp_ is the minimum value of *I*_raw_(τ,1) across values of τ. α_grp_ was then taken to be the maximum value of *I*_raw_(τ,*s*_pop_) − β_grp_ across values of τ and *s*_pop_. Thus, a value of 0 indicates the least information observed at any lag for the single most informative neuron, and a value of 1 indicates the greatest information observed at any lag for the largest population size tested. Results obtained using the unit order above were confirmed in a control analysis that randomly permuted the unit order before computing the information curves.

#### Testing effect of choice on noise correlation and f′ × f′ relationship.

We wanted to test whether the relationship between noise correlation (*r*_CCG_) and the product of tuning curve derivatives (*f*′ *× f*′) was dependent upon the correctness of the choice. Values were computed using a 500-ms-wide analysis window that was tiled across the Present RDS phase (Fig. 1*Aiii*) at 250-ms steps, such that windows at adjacent positions were overlapping each other by 50%. At each window position, a unique pair of *r*_CCG_ and *f*′ *× f*′ values was computed for each pair of neurons, each baseline disparity, and each combination of popout target location (IN or OUT of RF) and choice location (IN or OUT of RF). To visualize the dependence on choice, the Spearman correlation of *r*_CCG_ and *f*′ *× f*′ was computed across neuron pairs and baseline disparities at each window position, for each combination of target and choice location. The results are shown in Fig. 9. Each Spearman correlation value was classified as significantly different from 0 at a 5% level (Fig. 9, filled vs. empty circles, 2-tailed test).

We needed a way to verify the results shown in Fig. 9 without using repeated tests that might inflate the number of false positive results, such as an ANOVA. In the ideal experiment, one would have many more subjects than we did, in order to sample distributions of the *r*_CCG_ versus *f*′ *× f*′ Spearman correlations, for each trial condition and time step. One could then use an ANOVA to ask whether the correctness of choice could predict changes in the Spearman correlation.

With only two subjects, we had to devise an alternative approach. Theory predicts that there is a linear relationship between *r*_CCG_ and *f*′ *× f*′ (*Eq. 4* in Ref. 11), which is therefore proportional to the ratio of *r*_CCG_ over *f*′ *× f*′. In other words *r*_CCG_/*f*′*f*′ will be proportional to the measured correlation between *r_CCG_* and *f*′ × *f*′. Hence, we used *r_CCG_*/*f*′*f*′ as a way of quantifying the correlation of *r_CCG_* and *f*′ × *f*′. The advantage is that *r_CCG_*/*f*′*f*′ can be computed for each combination of neuron pair and disparity, for each combination of trial condition and time step, separately for each subject. This provides the sample distributions required to perform an ANOVA. Unfortunately, the distribution of the ratio of raw values was so strongly leptokurtic that it was inappropriate for use in an ANOVA test.

The following method (*Eqs. 31–33*) was applied in order to transform the ratios so that their distribution had one central peak, was relatively symmetric, and had tails that quickly asymptote, making them appropriate for use with an ANOVA. *r*_CCG_ and *f*′ × *f*′ values from each subject were grouped by window position, popout target location (IN or OUT), and type of neuron pair (within V1, within V4, between V1 and V4). For the *g*th grouping (1 unique combination of subject, window position, target location, and pair type), the *r*_CCG_ values were ranked (adjusted for ties; function *tiedrank*, MATLAB), and so were the *f*′ *× f*′ values. This transformed the two sets of values from group *g* into uniform distributions. Within group *g*, the *i*th combination of neuron pair and baseline disparity produced $q_{i,r_{\mathrm{CCG}}}$ and *q_i_*_,_*_f′f′_*, the ranked *r_CCG_* and *f*′ × *f*′ values, respectively. Each ranked value was then normalized:

(*31*) $$nq_{i,r_{\mathrm{CCG}}}=\frac{q_{i,r_{\mathrm{CCG}}}}{q_{\max,r_{\mathrm{CCG}}}}$$

(*32*) $$nq_{i,f^{\prime }f^{\prime }}=\frac{q_{i,f^{\prime }f^{\prime }}}{q_{\max,f^{\prime }f^{\prime }}}$$

where $q_{\max,r_{\mathrm{CCG}}}$ and *q*_max,_*_f′f′_* are the maximum ranks of each value, observed in group *g*. This resulted in sets of values that were uniformly distributed between 0 and 1. The log ratio of normalized ranks was taken:

(*33*) $${\log\;}_{10}\frac{nq_{i,r_{CCG}}}{nq_{i,f^{\prime }f^{\prime }}}$$

Critically, the log ratio preserved the relative position between correct (Target IN and Choice IN or Target OUT and Choice OUT) and incorrect (Target OUT and Choice IN or Target IN and Choice OUT) data; note that incorrect Target OUT and Choice OUT trials were not used. The output of *Eq. 33* was used in an all-ways ANOVA (1 per subject) testing the effect of experiment day, window position, type of neuron pair, and correctness of choice that was further examined with Tukey–Kramer’s multiple-comparisons test.

#### Detect probability using optimal projection of population responses.

To test the possible effect of differential correlations on perceptual choice, we needed a way to obtain a trial-by-trial estimate of differential correlation. This was done following the technique of Ref. 13. Let *n* be a column vector of firing rates measured from the simultaneous responses of *N* neurons on a single trial. The mean value of the firing rates in response to baseline disparity *d* was μ*_d_* = E[*n*|*d*]. For any *d* ∈ *B*, where *B* is the set of baseline disparity values, μ*_d_* was estimated using the sample mean calculated from an analysis window −200 to 0 ms before the start of the popout event. After the start of the popout event, the binocular disparity of the target RDS was *d*_p_ = *d*_b_ + *s*, where *d*_b_ ∈ *B* and *s* is one of several nonambiguous disparity steps (±0.05°, ±0.1°). For any *d*_p_ ∉ *B*, ${\text{μ}}_{d_{p}}$ was estimated from the measured disparity tuning curves using linear interpolation. The signal vector was defined as $\text{Δ}f_{d_{b}d_{p}}={\text{μ}}_{d_{p}}-{\text{μ}}_{d_{b}}$, describing the expected change in firing rate between responses to the baseline and popout disparities. *Equation 28* was used to define the *N *×* N* covariance matrix Σ*_d_*(τ) for each *d* ∈ *B*, using the same −200 to 0 ms window. Note that although μ*_d_* and Σ*_d_*(τ) were always measured from the −200 to 0 ms window, the population responses *n* were measured from a set of different analysis window positions using different τ values, so that the analysis could examine the potential interaction of differential correlation and choice at different points in time, for different temporal scales of noise correlation.

To discriminate the baseline population response from the popout response, the optimal weighted sum of firing rates will use weights *w*_opt_ ∝ Σ^−1^Δ*f*, similar to the solution for linear discriminant analysis. Hence, the weights used on a given trial with baseline disparity *d*_b_ and popout disparity *d*_p_ were defined as $w_{d_{b}d_{p}}\left(\text{τ}\right)={\left({\text{Σ}}_{d_{b}}^{-1}\left(\text{τ}\right)\text{Δ}f_{d_{b}d_{p}}\right)}^{tr}$. Thus, the optimal set of weights were tailored to discriminate the baseline and popout disparity on each trial. The raw projection $y_{\mathrm{raw}}=w_{d_{b}d_{p}}n$ was normalized:

(*34*) $$y\left(\text{τ}\right)=\frac{w_{d_{b}d_{p}}\left(\text{τ}\right)\left(n-{\text{μ}}_{d_{b}}\right)}{w_{d_{b}d_{p}}\left(\text{τ}\right)\text{Δ}f_{d_{b}d_{p}}}$$

such that E[*y*|*d*_b_] = 0 and E[*y*|*d*_p_] = 1; that is, the mean value of the projection was 0 for responses to the baseline disparity and 1 for responses to the popout disparity. Because the optimal linear weights were used, the linear combination of population responses could mitigate any nondifferential noise correlations. Thus, any remaining variance in *y* was possibly due to information-limiting, differential correlations.

The optimal projection of population responses, *y*, was measured on each trial for each composition of neural population (V1 units only, V4 units only, all V1 and close V4 units). Hence, we could use *y* to predict the behavioral choice using signal detection theory to compute a measure analogous to choice probability (40). After grouping trials by target location (IN or OUT of the RF), we labeled choices IN to the RF (correct choice when target IN, incorrect when target OUT) as “true positives” and choices OUT of the RF (correct when target OUT, incorrect when target IN) as “false positives” before computing the area under the receiver operating characteristic curve (AROC; see *Eq. 38* below).

We pooled *y* across experimental days and across subgroups of trials, separately per subject. Pooling was necessary because of the large number of possible subgroups of trials. With 13 baseline disparities, 5 popout step sizes, 4 target locations, and 4 choice locations there were 13 × 5 × 4 × 4 = 1,040 possible subgroupings of trials. This is comparable to the number of trials collected on a single experimental day; many subgroups had no trials at all, and few had more than one. Our normalization technique allowed us to pool data across subgroups of trials and also across experimental days; the latter is justified because the same cortical sites were recorded each day thanks to the chronic Utah arrays. Thus, we were able to build up a sufficient number of trials to obtain the statistical power required by the nonparametric methods that we used to evaluate *y*.

In total, we pooled 1,168 choice IN (197 choice OUT) trials for *M135* and 819 (285) for *M138* when the popout target RDS was IN the RF and 982 (2,427) for *M135* and 791 (1,918) for *M138* when the target was OUT; this was an ample number of trials to effectively compute AROC (40). The significance of AROC values was estimated by computing bootstrap 95% confidence intervals (CIs), obtained from 20,000 bootstrap samples by the percentile method. We called this value “detect probability” [DP(τ) using *y*(τ)] because the animals had to detect the location of any disparity change without reporting the direction (“near” or “far”) of the change. However, this DP is not strictly the same as the original DP of Cook and Maunsell (41), whose task used temporal rather than spatial uncertainty.

Here, DP is the probability that an ideal observer could predict the subject’s choice (IN or OUT of RF) using the optimal projection of the population response, *y*. If DP > 0.5, then *y* tended to be more positive (i.e., closer to popout disparity) before choices IN to the RF. DP < 0.5 shows that *y* was more negative (i.e., further from popout disparity) before choices IN. DP = 0.5 is the chance level, showing no discernible differences in the distributions of *y* for choice IN and choice OUT. In other words, DP is a measure of the probabilistic correlation between *y* and the choice that was made, as the same value of *y* could be observed before either choices IN or OUT of the RF.

#### A model V4 output neuron can attenuate differential noise.

We modeled a theoretical V4 output neuron in order to test whether a simple mechanism could attenuate differential noise that was present upstream in V1 and V4. Thus, the model unit had only two afferent inputs, a V1 unit and a V4 unit (see Fig. 11*A*). There were two chief parameters of the model, the signal correlation of the V1/V4 input pair and the width of a window used to integrate their responses. V4 output responses were predicted by subtracting a weighted version of the V1 input from the V4 input. V4 output neurons were modeled for all recorded V1/V4 pairs as afferent inputs and for window widths of 20, 40, 80, and 160 ms for Initial responses or 100, 200, 400, and 800 ms for Steady state responses.

The V1 input weight (*w* in Fig. 11*A*) should reflect the magnitude of noise shared by the afferent pair that was not explained by the random sampling of disparity values from trial to trial. To measure it, we used a simple generalized linear model (GLM) to predict the spike count of the V4 input from its disparity tuning curve and the spiking of the V1 input, using the ln link function:

(*35*) $$\text{ln}\;n_{\text{V}4}\left(m\right)=c_{0}+c_{1}G_{\text{V}4}\left(d_{m}\right)+c_{2}n_{\text{V}1}\left(m\right)$$

*n*_V4_(*m*) is the spike count of the V4 input unit on trial *m*, *G*_V4_ is the Gabor function that best fit the V4 input’s tuning curve, *G*_V4_ was evaluated at the disparity value *d_m_* used on trial *m*, and *n*_V1_(*m*) is the spike count of the V1 input unit on trial *m*. The GLM was used to fit (function *glmfit*, MATLAB) three parameters: the baseline spike count (*c*_0_), the amount of disparity signal (*c*_1_), and the amount of shared variance between the afferent pair (*c*_2_). The Gabor term was important, as it helped to push *c*_2_ toward accounting for shared residual variance rather than shared signal.

Once obtained, we predicted the V4 output responses with

(*36*) $$n_{\text{V}4,\mathrm{OUT}}\left(m\right)=n_{\text{V4}}\left(m\right)-\exp\;\left(c_{2}n_{\text{V1}}\left(m\right)\right)+\beta$$

where *n*_V4,OUT_(*m*) is the V4 output spike count on trial *m*. Thus, the V4 output is the V4 input minus the weighted V1 input. β is a constant term that restores the baseline value of the modeled V4 output disparity tuning curve to match that of the V4 input. Any negative values were capped at zero. This produced V4 output spike counts with a dynamic range similar to the V4 input. The exponential function was used to transform the scaled V1 response because the logarithmic link function was used in the GLM.

To avoid overfitting, we applied *Eqs. 35* and *36* to different halves of the trials in a twofold, cross-validation procedure. Trials were divided into even- and odd-numbered groups. Thus, the training and test sets had a similar size and composition, balancing the need to train the GLM against the need to accurately compute AROC (see below). One group of trials was used to train the model with *Eq. 35*, and then the other group was used to evaluate it with *Eq. 36*. The process was repeated a second time after reversing the roles of each group. AROC was used to evaluate the disparity selectivity of the V4 output responses for each test set, yielding two estimates per condition that were averaged together.

The training, testing, and evaluation procedure was applied to the empirical data, in which the intercortical noise correlations were intact (Fig. 11*C*, thick solid line, “Not shuffled”). However, it was necessary to determine the degree to which any change in disparity selectivity of the V4 output responses was due to the combined disparity information signaled by the afferent pair. Therefore, the procedure was applied a second time to data in which the V1 responses were shuffled between trials. Shuffling was done by swapping responses between trials with the same disparity, so that the disparity selectivity of both the V1 and V4 input units was preserved but the noise correlations were destroyed (Fig. 11*C*, thin solid line, “V1 tuning kept”). A third evaluation was done using shuffled data in which responses were shuffled across all trials, regardless of disparity, so that the noise correlations and V1 disparity selectivity were abolished (Fig. 11*C*, dashed line, “All trials shuffled”).

We evaluated summation of the V1/V4 inputs with integration windows of different widths (Fig. 11*D*). This was accomplished by subdividing each analysis window (Initial or Steady state) into a set of smaller, contiguous windows that were each Δ*t* ms wide. Spike counts were obtained, and *Eqs. 35* and *36* were applied separately for each subdivision; that is, an independent set of GLM parameters was trained and tested for each subdivision of the analysis window. The V4 Gabor function was scaled to produce the spike count expected in a window of size Δ*t* ms. Before AROC was computed, the V4 output spike counts were summed back together across subdivisions:

(*37*) $$n_{\text{V4,OUT}}\left(m\right)=\overset{W}{\underset{\text{ω}=1}{\sum }}n_{\text{V4,OUT}}\left(m,\text{ω}\right)$$

where *n*_V4,OUT_(*m*,ω) is the V4 output spike count obtained on trial *m* in the ωth subdivision of the analysis window, out of *W* subdivisions with a width of Δ*t* ms.

AROC was used to evaluate the model instead of DMI. In computing DMI nonparametrically, it is possible that the true information was underestimated as a result of discretizing the data through binning, analogous to the pixilation of a low-resolution photograph. Therefore, we used a continuous method based on signal detection theory (42) that has previously served to quantify the sensitivity of single neurons to different stimulus values (5). We used a similar approach to estimate disparity sensitivity, in which a receiver operating characteristic (ROC) curve is built by labeling one set of spike counts as true positives and another as false positives. If the true positive spike counts were from trials with one disparity value and the false positives were from trials with another, then the area under the ROC (AROC) becomes the probability that an ideal observer could correctly classify the true positive spike counts. This leads directly to a measure of how well the neuron can discriminate the two tested disparities. A chance value of AROC = 0.5 means that the two spike-count distributions were indistinguishable. Values of 0 or 1, however, indicate perfect separation of the distributions, with intermediate values of AROC indicating different amounts of overlap. We calculated AROC using trapezoidal integration of the true- and false-positive cumulative distribution functions (*F*_T_ and *F*_F_, respectively):

(*38*) $$\text{AROC}=\frac{1}{2}\overset{V}{\underset{i=1}{\sum }}\left[F_{\text{F}}\left(x_{i}\right)-F_{\text{F}}\left(x_{i-1}\right)\right]\left[F_{\text{T}}\left(x_{i}\right)+F_{\text{T}}\left(x_{i-1}\right)\right]$$

where *x_i_* ∈ {*x*_1_, *x*_2_, *x*_3_, … *x_V_*} is the ordered (ascending) set of *V* unique spike counts from either the true- or false-positive distribution and *F*_T_(*x*_0_) = *F*_F_(*x*_0_) = 0. The absolute difference of AROC from chance is a measure of separation between two spike count distributions regardless of which was labeled as the true or false positives:

(*39*) |AROC|=|AROC−0.5|

|AROC| was computed for each of the 78 unique pairs of tested disparity values. With 4,598 V1/V4 afferent pairs, we predicted a total of 358,644 AROC values for the model V4 output neurons. The difference in V4 output and V4 input disparity selectivity is shown in Fig. 11*C*.

#### RF mapping.

To measure RF separation, we performed an independent quantification of the positions and sizes of the V1 and V4 RFs. RF position was mapped in separate experiments by placing a small random dot stereogram (RDS) at randomly selected positions on a grid of possible locations while the animal maintained fixation; the RDS position changed between trials. The size of the RDS was fixed (2.5° diameter) and the binocular disparity was always 0°. Thus, we mapped the average multiunit firing rate for each electrode across RDS positions. We quantified each RF map by fitting it (least squares; function *lsqcurvefit*, MATLAB) with the two-dimensional isotropic Gaussian:

(*40*) $$f\left(x,y\right)=c_{0}+c_{1}e^{-\left(\frac{{\left(x-c_{2}\right)}^{2}+{\left(y-c_{3}\right)}^{2}}{2c_{4}^{2}}\right)}$$

where *f*(*x*,*y*) is the average firing rate at the point (*x*,*y*) degrees from the center of gaze, *c*_0_ is the baseline rate, *c*_1_ is the amplitude, (*c*_2_,*c*_3_) is the mean, i.e., center of the Gaussian, and *c*_4_ is the standard deviation. The center and standard deviation were taken as measures of the RF center and size.

The Gaussian fits were reasonably accurate (median coefficient of determination, i.e., *r*^2^; *M135* V1 = 0.875, 95% boot CI [0.788, 0.881]; *M135* V4 = 0.650, [0.517, 0.813]; *M138* V1 = 0.966, [0.962,0.968]; *M138* V4 = 0.862, [0.739, 0.926]), exhibiting the expected, fundamental relationship between RF eccentricity and size (all RFs: Spearman correlation *M135* = 0.829, *P* → 0; *M138* = 0.634, *P* = 3.351e–9).

The RF separation of pairs of neurons was defined by computing the Euclidean distance between RF centers, in degrees of visual field. For neurons *i* and *j* (*i *≠* j*), RF separation was

(*41*) $${\text{RF}}_{\text{separation}}=\sqrt{{\left(x_{j}-x_{i}\right)}^{2}+{\left(y_{j}-y_{i}\right)}^{2}}$$

where (*x_i_*,*y_i_*) is the center of the Gaussian fit to the RF map of the *i*th neuron, similarly for the *j*th neuron.

Each V4 unit was classed by its average RF separation to all simultaneously recorded V1 units. V4 RFs were considered to be “close” to (<7.99°) or “distant” from (>7.99°) V1 RFs. The criterion separation (7.99°) made the pooled number of close V1/V4 pairs in *M135* the same as the number of distant V1/V4 pairs in *M138*. Thus, the smaller group from each animal had comparable statistical power. The average number of close V4 units across experimental days was 5 [standard deviation = 0.8 units] in *M135* and 22 [3.3] in *M138*; the average number of distant V4 units was 13 [2.8] in *M135* and 3 [0.8] in *M138*. Hence, the average number of pairs of V1 and close V4 neurons was 66 [31.0] in *M135* and 749 [161.8] in *M138*; the average number of pairs of V1 and distant V4 neurons was 134 [63.0] in *M135* and 98 [31.7] in *M138*.

#### Correlation coefficients.

Typically, the correlation between two variables was measured using the Spearman correlation coefficient because our data were often not Gaussian distributed. This was especially true when considering the product of derivatives, which had a typical product distribution with a strong kurtosis and prominent peak at zero (see Fig. 6). Being a nonparametric method, the Spearman coefficient was mainly used in situations where the data were pooled, to provide the necessary statistical power. However, we switched to using the Pearson correlation coefficient for some of the control analyses in which there was less data and, consequently, a more powerful parametric method was needed. For instance, some of the main Spearman correlation analyses that pooled the data across sessions were then verified, using example sessions and Pearson correlation.

Both types of correlation were calculated using MATLAB’s *corr* function. Thus, Pearson correlation *P* values were found with a Student *t* distribution for a transformation of the correlation, and Spearman correlation *P* values were found using either the exact permutation distributions (small sample sizes) or large-sample approximations. Furthermore, *P* values for two-tailed tests were obtained by doubling the more significant of the two one-tailed *P* values.

#### Goodness of fit.

RF Gaussian and tuning curve Gabor fits were evaluated with the coefficient of determination, i.e., *r*^2^.

#### Bootstrap confidence intervals.

Unless otherwise stated, 95% bootstrap confidence intervals were found with the bias corrected and accelerated percentile method (function *bootci*, MATLAB) with at least 1,000 bootstrap samples.

## RESULTS

We trained two rhesus macaques (*M. mulatta*) to observe four patches of dynamic random dot stereograms (RDSs) and perform an odd-one-out task (Fig. 1), in which they detected which one of the four patches displayed a change in binocular depth. Each RDS was configured as a central circular patch, whose binocular depth varied from trial to trial, and a surround region of dots located in the fixation plane of the monkeys’ eyes. In the “Present RDS” phase (Fig. 1*Aiii*), all four stimuli had the same depth for the central patch, which was chosen from 1 of a set of 13 different values. The “popout” phase (Fig. 1*Aiv*) presented the change in depth, which was applied to the center of one of the four RDS stimuli. That patch became the target for a saccade to indicate a correct choice and earn a reward; a saccade to any of the other three RDS was incorrect and earned no reward.

The monkeys’ behavior indicated attention to the RDS stimuli in the Present RDS phase, as they were better at reporting the location of the disparity step change as it grew in magnitude (Fig. 1*B*, % correct), while their reaction times (RTs) decreased. On catch trials with no change in the RDS, the animals had to guess, so their accuracy was at chance levels (Fig. 1*B*, *left*, dashed line) and their RTs were longest (Fig. 1*B*, *right*). Figure 1*C* shows the relationship between percent correct as a function of RT using a 50-ms moving window, grouped by magnitude of the popout disparity change. Correct decisions are broadly within the window of 100–400 ms after popout.

Neural activity in the dorsal, gyral portions of V1 and V4 was recorded with two 64-channel (8 × 8; 0.4-mm spacing) Utah array electrodes (1 array per area) implanted chronically in the left hemisphere. The electrode placement meant that the recorded neurons had receptive fields (RFs) that were stimulated by the lower right RDS (Fig. 2*A*). An automatic spike sorting method was used to identify action potentials followed by manual checking (see methods for details.) As with other work with multichannel probes (21, 33), analyses were based on both single units and multiunit clusters, and the term “unit” refers interchangeably to either.

Variations between neuronal RFs with respect to spatial location and preference for binocular depth, dot size, dot contrast, and other dimensions of the RDS stimulus mean it is impossible to simultaneously achieve optimal stimulation of every unit recorded by the arrays. We therefore focused on varying these parameters to ensure that there was significant coactivation of the V1 and V4 units (Fig. 2, *D* and *E*). To validate the coactivations of neuron pairs, we applied entry criteria, such that each unit in the pair had to show statistically significant tuning to disparity (1-way ANOVA at the 1% level) and fire at least 5 spikes per second to its preferred disparity. For all the recordings reported here, the animals were performing the stereo depth task, which requires allocation of attention to the four RDS patches.

We made separate measurements of RF location and stimulus preferences in preliminary experiments in each monkey. These measurements demonstrated that there was scatter in the positions of the RF centers of the units, both within and between cortical areas (Fig. 2*A*). We address how this separation of RF centers may have influenced our results below. Here, we confine ourselves to noting that the combination of electrode placements and adjustment of the configuration of the RDS stimulus resulted in common activation of pairs of V1 and V4 units by the center portion of the same RDS. We focus on the activations of units from the “Hold fixation” and Present RDS phases of the trial (Fig. 1, *Aii* and *Aiii*, and Fig. 2, *D* and *E*), before the arrival of the discrimination stimulus itself.

### V1 and V4 Units Were Selective for Binocular Disparity

The V1 and V4 neural signals that we recorded are able to support task performance. Disparity tuning curves are shown in Fig. 2*B* for example V1 (circles, gold line) and V4 (plus sign, blue line) units. The solid lines are the best-fitting Gabor functions (*Eq. 21*; Ref. 34), which describe the response profiles as a function of disparity. To quantify a unit’s contribution to the estimation of stimulus disparity, we computed how much information the firing of the neuron could contribute to the identification of the currently displayed disparity of the RDS, called the disparity mutual information (DMI; *Eq. 8*; Fig. 2, *C* and *E*). This measure correlated well with a previously established measure of selectivity, the disparity discrimination index (DDI; Ref. 34), for the V1 (Spearman correlation, Initial, *r *=* *0.976, *P* = 9.559e−130) and V4 (*r *=* *0.953, *P* = 4.705e−111) neurons.

The neurons in V1 and V4 that responded most to the onset of the stimulus were also the most selective for disparity, as the peak firing rate and DMI (in the 50 to 200 ms window) were well correlated (Fig. 2*C*; V1 in gold, Spearman *r *=* *0.609, *P* → 0; V4 in blue, *r *=* *0.248, *P* = 2.663e−4). Low-firing neurons were relatively poor at encoding disparity compared with highly active neurons.

We searched for the time point at which the units became selective for disparity by convolving the spike trains with a causal exponential kernel (20 ms time constant) to obtain the firing rate and DMI time series for each unit. The average firing rates (Fig. 2*D*; V1, gold; V4, blue) had a transient burst of activity in response to the onset (black dotted line) of the RDS, which then settled down to a steady rate. As expected, V1 began responding to the RDS before V4 and V1 neurons showed selectivity for the binocular disparity slightly earlier than V4 neurons.

Firing rates in both areas were selective for binocular disparity almost as soon as the units began responding to the RDS, with the average DMI showing a time course similar to the changes in the firing rate (Fig. 2*E*). We verified the link in timing between the DMI and the peak firing for each unit by finding the Spearman correlation of firing rate and DMI, matched by time bin, in an 800-ms window starting 50 ms after the RDS onset, i.e., we took the zero-lag cross correlation of the firing rate and DMI time series. The result was significantly positive for V1 (mean *r *=* *0.192, right-tailed *t* test *P* = 3.339e−22) and V4 (*r *=* *0.245, *P* = 7.819e−31), showing that individual units were more selective for disparity at moments in the trial when their firing rate was higher.

We partitioned each trial into three consecutive windows for further analysis (Fig. 2, *D* and *E*). The Spontaneous window (Fig. 2, *D* and *E*, leftward arrow) captured neural responses during the Hold fixation phase (Fig. 1*Aii*) of the trial, in which the monkeys held their gaze on the fixation target before the arrival of RDS signals in V1. The response to the onset of the RDS is captured by the Initial window (Fig. 2, *D* and *E*, thick bar and black solid lines, marking 50–200 ms after stimulus onset), which shows a transient burst of firing and rise in the DMI. The responses to the RDS for the remainder of the Present RDS phase of the trial are captured by the Steady state window (Fig. 2, *D* and *E*, rightward arrow; Fig. 1*Aiii*). Within-unit comparisons showed that disparity selectivity (DMI) was stronger in the Initial than in the Steady state window for V1 (mean paired difference = −0.577 bits/s, left-tailed *t* test *P* = 1.294e−12) and V4 (−0.220 bits/s, *P* = 6.827e−11); in part, these differences reflect the greater firing rates in the Initial window.

### Information-Limiting Correlations

**Figure 3.**
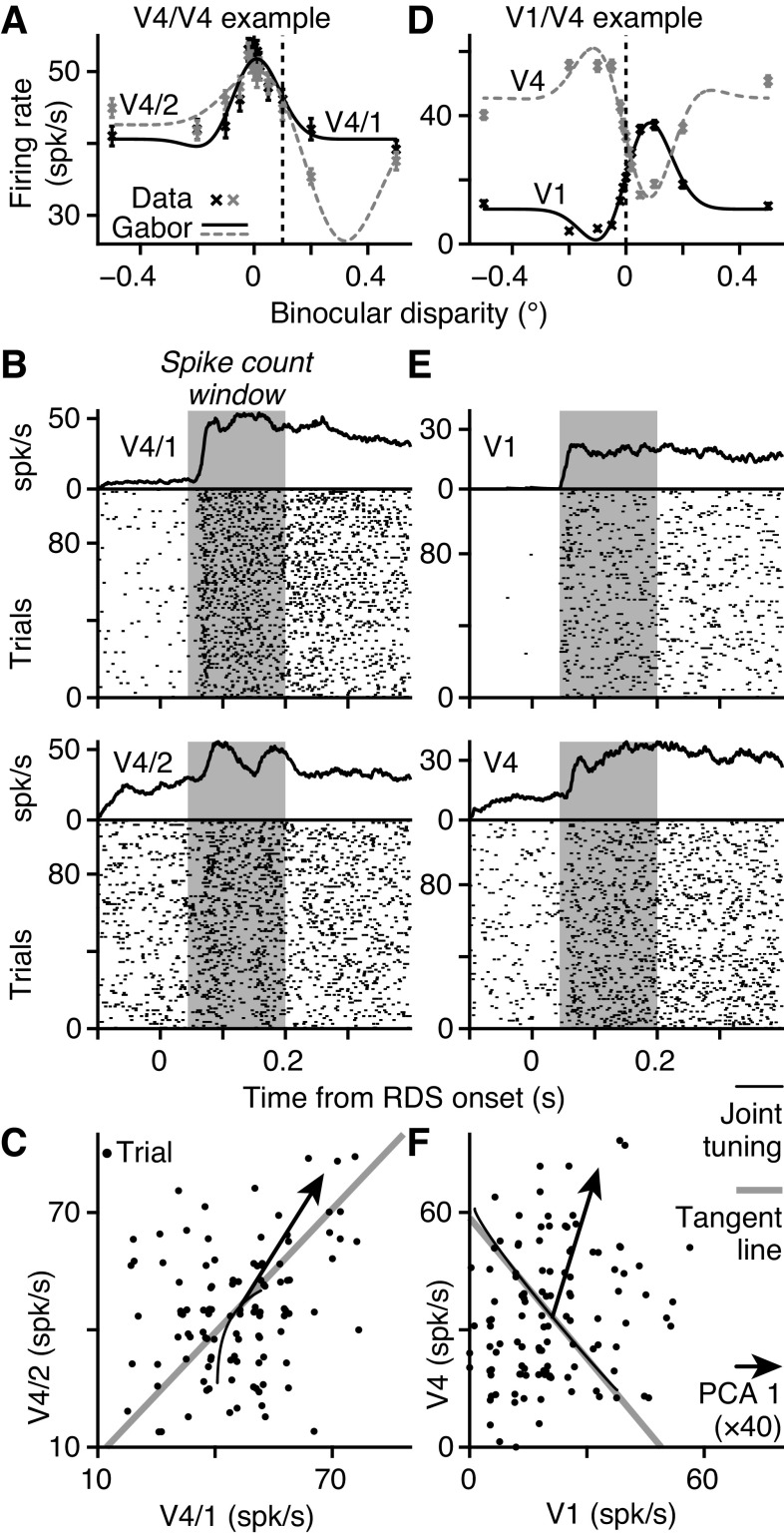
Alignments of noise correlations and disparity tuning. *A*: disparity tuning curves of pair of extrastriate area V4 neurons with positive signal correlation (*r*_signal_ = +0.851); V4/1 (black) and V4/2 (gray), crosses and SE show average firing, lines show best-fitting Gabors (V4/1 *r^2^* = 89%, V4/2 *r^2^* = 93%). Vertical dashed line at +0.1° shows trials (*N* = 108) examined in *B* and *C*. *B*: raster plots and average firing rates for V4/1 (*top*) and V4/2 (*bottom*); random dot stereogram (RDS) onset at *t* = 0; for 108 trials at baseline disparity +0.1°; shading shows time window for firing rates in *C*. *C*: dots show joint firing rates; slight jitter applied for visualization; noise correlation, spike count correlation (*r*_SC_) = 0.241, *P* = 0.012. Tangent line (gray) shows joint tuning curves at +0.1°; arrow shows first principal component analysis (PCA) component (×40, for visibility) of joint responses. Thin black line shows local neighborhood of the joint tuning curve. *D–F*: same as *A–C* but for an example primary visual cortex (V1)/V4 pair (V1 black in *D*, *r^2^* = 98%; V4 gray in *D*, *r^2^* = 94%) with negative signal correlation (*r*_signal_ = −0.982). Trials with baseline disparity of 0° (*D*, black dashed, *N* = 116) yield significant positive noise correlation (*F*, *r*_SC_ = 0.203, *P* = 0.029). *A–C*: data from pair number M138.336.94/147 in our database. *D–F*: data from pair number M138.336.57/87. spk, Spikes.

If the sensory discrimination task performed by the monkeys is supported by a linear computation across pools of active neurons, then a special class of noise correlation, differential correlation (11), is potentially a fundamental limit on the information that is encoded by the sensory pool. Figure 3, *A*–*C*, show how differential correlations could limit information that is retrievable from a pair of neurons whose activities were simultaneously recorded in V4 during task performance. The pair had similar tuning for binocular disparity (i.e., 0 < *r*_signal_; Fig. 3*A*). Both units had a common negative slope in their tuning curves around 0.1° (Fig. 3*A*, black dashed line), where they were sensitive to the same small changes in disparity. Adding the signals from these two neurons potentially improves sensitivity, but this depends on the noise distributions.

Joint responses to multiple presentations of the 0.1° disparity stimulus were obtained by counting spikes in the Initial window (Fig. 3*B*, gray). At 0.1°, the pair had a positive noise correlation (Fig. 3*C*). Critically, the principal component (Fig. 3*C*, arrow) of the covariance was nearly parallel to the tangent line (gray) of the tuning curves (black) at 0.1°. To see the consequence of this, consider a linear discriminator that classifies the joint responses as one of two disparities on either side of 0.1°. If the decision boundary is perpendicular to the tangent line (Fig. 3*C*, gray), then noise correlation (arrow) will make the joint responses jump across the decision boundary on some trials, causing classification errors (10). Put simply, correlated noise would mimic true changes in the stimulus, limiting the sensitivity of this pair.

This outcome contrasts with the responses of a second pair (Fig. 3, *D*–*F*) recorded simultaneously from V1 and V4. As before, both units had steep, overlapping slopes in their tuning curves (Fig. 3*D*). Unlike the V4/V4 pair, the V1 and V4 units were selective for opposite disparities (*r*_signal_ < 0). Spike counts from both units in response to a disparity of 0° (Fig. 3*D*, black dashed line) were obtained as before (Fig. 3*E*), and the joint responses were examined (Fig. 3*F*). Although this pair has a negative signal correlation, they have a positive noise correlation (*r*_SC_ = 0.203; *P* = 0.029): the noise correlation does not align to the tangent [noise correlation first principal component PCA1 at 72.8° (95%CI: 58.5°, 85.3°)]. In this case, correlated noise would be less likely to leak across the decision boundary.

### *r*_CCG_ Measures Noise Correlation at Different Timescales

**Figure 4.**
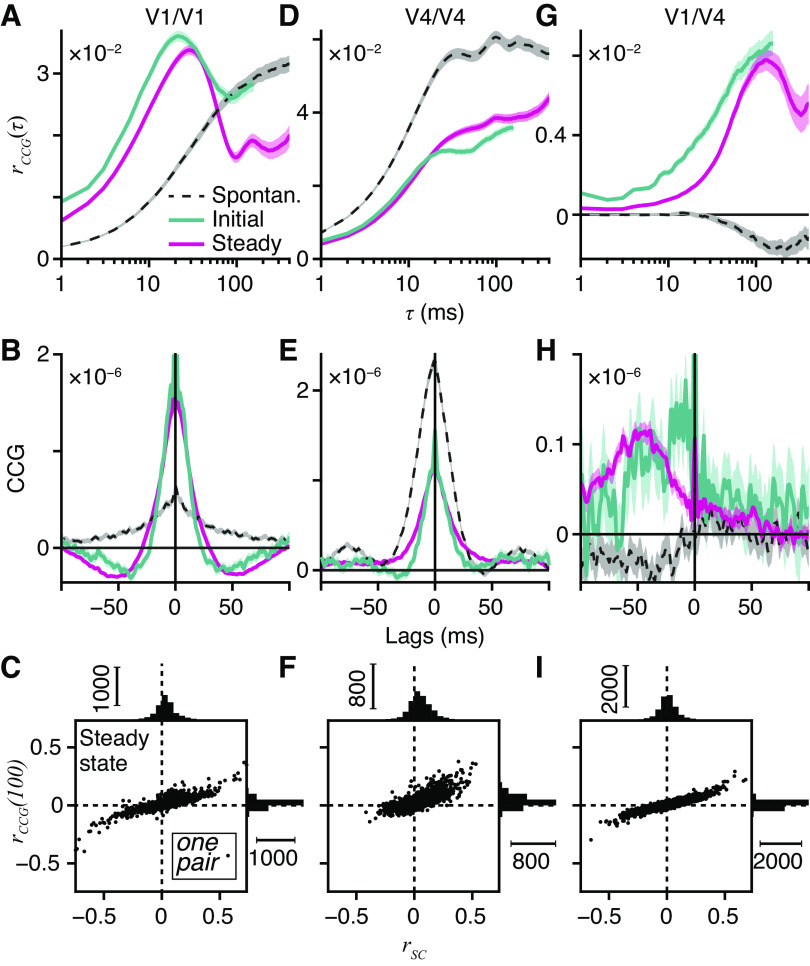
*r*_CCG_ measures noise correlation at different timescales. *A*: average *r*_CCG_ as function of temporal scale τ for all primary visual cortex V1/V1 pairs’ (*N* = 2,419) Spontaneous (dashed black), Initial (teal), and Steady state (magenta) responses (see Fig. 2*D*); shading shows SE. *B*: as *A* but has average V1/V1 cross correlograms (CCGs). *C*: scatterplot of Steady state *r*_CCG_ at τ = 100 ms vs. spike count correlation (*r*_SC_). Marginal distributions show each metric separately. *D–F*: same as *A–C* but for extrastriate area V4/V4 pairs (*N* = 2,226). *G–I*: same as *A–C* but for V1/V4 pairs (*N* = 4,598). spk, Spikes; V1, primary visual cortex; V4, extrastriate area.

Noise correlations (*r*_SC_, *Eqs. 10* and *11*) are captured experimentally by setting up a window of time in which to count the number of spikes in response to repeated presentations of the same stimulus. In terms of timing, this encompasses the synchronous firing of spikes with millisecond precision up to long-timescale cofluctuations in spike numbers (16, 17, 43). Measurement of spike count correlation confounds these slow and rapid correlations, depending on the size of the counting window that is used. Another, time-based measure, *r*_CCG_ (*Eq. 17*), is used to reveal noise correlations at different temporal scales (15). This measure, *r*_CCG_, is the symmetric integral over time lags of the spike train cross correlation and reveals the timing relationships between correlated spikes as well as the strength of the correlation. The *r*_CCG_ measure converges on *r*_SC_ as the width (τ) of the integration reaches that of the time window used to count the spikes for *r*_SC_ and to provide spike trains for *r*_CCG_. We asked whether information-limiting correlations occupy a limited set of temporal scales by using *r*_CCG_ to examine noise correlations within cortical areas V1 and V4 and between those two cortical areas (Fig. 4).

Within V1 (Fig. 4*A*), the timescale of noise correlations changed as the trial progressed. Before the RDS appeared, correlations occupied a slow timescale, being strongest on the order of hundreds of milliseconds (Fig. 4*A*, dashed line). The time window for correlations became tighter after RDS onset, peaking at τ = 20 ms; *r*_CCG_(τ = 20 ms) was greater in the Initial window (Fig. 4*A*, teal line) than the Spontaneous window (mean paired difference = 2.141e−2, paired *t* test *P* = 1.592e−124). Even in steady-state responses to the RDS (Fig. 4*A*, magenta line), the slower correlations were weaker, with a drop in *r*_CCG_ (τ = 100 ms) from the Spontaneous window to the Steady state window (−1.092e−2, *P* = 1.005e−21).

These changes in *r*_CCG_ with timescale are reflected in the shapes of the V1-V1 cross correlograms (CCG, *Eq. 19*; Fig. 4*B*). In the period of Spontaneous activity, the CCG was positive and had a broad peak, coinciding with the steady increase of Spontaneous *r*_CCG_ as τ increased. In contrast, the Initial and Steady state CCGs had narrow positive peaks flanked by negative side lobes, showing that the majority of correlated spikes occurred within tens of milliseconds of each other. This matched the peaks in Initial and Steady state r_CCG_ at τ = 20 ms.

Unlike V1, correlations within V4 (Fig. 4*D*) began saturating at τ = 20 ms in all three analysis windows. V4 correlations were also stronger in the Spontaneous window, with *r*_CCG_(τ = 20 ms) dropping in the Initial window (−2.188e−2, *P* = 9.053e−105) and then changing little from the Initial to Steady state windows (9.464e−4, *P* = 0.041). These differences appeared in the V4 CCGs (Fig. 4*E*) in which the Initial and Steady state CCGs were mainly attenuated versions of the Spontaneous CCG.

In marked contrast, the correlations between V1 and V4 (Fig. 4*G*) saturated at τ = 100 ms. Although these between-area correlations are weaker, it is important to understand that they were significantly positive during the stimulus presentation [mean *r*_CCG_(100 ms); Initial = 8.075e−3, right-tailed *t* test *P* = 1.512e−43; Steady state = 7.398e−3, *P* = 4.088e−61; Spontaneous = −1.286e−3, *P* = 1]. There was no measurable change in the V1/V4 correlations from the Initial to the Steady state window (mean difference = −6.767e−4, paired *t* test *P* = 0.159). The V1/V4 CCGs (Fig. 4*H*) reveal a consistent delay, whereby V1 spikes tended to occur up to 50 ms earlier than the associated V4 spikes; the significance of *r*_CCG_(100 ms), which integrates the entire area under the negative peak, suggests that this trend is also significant.

Bair and colleagues (15) established an important property of *r*_CCG_, which is that it provides a lower-variance estimate of short-term correlations compared with *r*_SC_. The parameter τ can be adjusted so that *r*_CCG_(τ) integrates only the central peak of the CCG and discards the longer time course components. These longer time course components are captured by *r*_SC_ but often add variance to that estimate. This principle is illustrated for the Steady state condition by a direct comparison of *r*_CCG_(100 ms) and *r*_SC_ in V1 (Fig. 4*C*), in V4 (Fig. 4*F*), and between areas (Fig. 4*I*). There was a strong, positive Spearman correlation of *r*_CCG_ and *r*_SC_ in all cases (V1 *r *=* *0.750, V4 *r *=* *0.772, V1/V4 *r *=* *0.807, all *P* → 0), showing that they measured a common source of correlation. However, the marginal distributions plotted at the top and right-hand sides of Fig. 4, *C*, *F*, and *I*, show that the variation (standard deviation) of *r*_SC_ (σ_SC_) was greater than that of *r*_CCG_(100 ms) (σ_CCG_) for all three cases (V1, V4, V1/V4; see Table 1). Hence, the Pitman’s *r*_SD_ test for comparing variances of correlated variables (44) shows that *r*_CCG_ is a more accurate measure of short-term correlations than *r*_SC_.

**Table 1.** Comparison of scatter in estimates of r_SC_ and r_CCG_(100 ms)

Cortical Area	σ_SC_	σ_CCG_ (τ = 100 ms)	Pitman’s *r*_SD_
V1	0.135	0.044	0.830
V4	0.112	0.056	0.766
V1/V4	0.112	0.030	0.886

Each *r*_SD_ has a *P* value approaching 0. *r*_CCG_, Time-based noise correlation; *r*_SC_, spike count correlation; σ_SC_, standard deviation of *r*_SC_; σ_CCG_, standard deviation of *r*_CCG_; V1, primary visual cortex; V4, extrastriate area V4.

### *r*_CCG_ Control Analyses

A drawback of using chronic Utah arrays is that any duplication of units between recording days might artificially boost the statistical significance of the results when the data are pooled across days. Another issue is that we had no prior reason to choose any specific values of τ for our analysis; the value of τ used in each analysis was chosen empirically. Therefore, we selected an example recording day for *M135* (*day 3* of *6*) and *M138* (*day 2* of *4*) because these yielded the largest number of V1/V4 pairs that had close RFs. In addition, we defined a standard set of τ values that were proximal to the *r*_CCG_ saturation points (Fig. 4, *A*, *D*, and *G*): τ = 20 ms when examining V1-only or V4-only sets of units, whereas τ = 100 ms was used to examine combinations of V1 and V4 units. These example days and standard timescales are used as a consistent benchmark in control analyses that verify our major results.

We began by confirming that *r*_CCG_ was significantly greater than zero (right-tail, 1-sample *t* test, *P* ≤ 3.528e−4) for each separate combination (2 subjects × 2 windows × 3 pair types = 12) of example day, Initial or Steady state window, and type of neuron pair (all available pairs per day) at the standard values of τ.

### Noise Correlations Varied with Disparity Tuning, Temporal Scale, and Time

**Figure 5:**
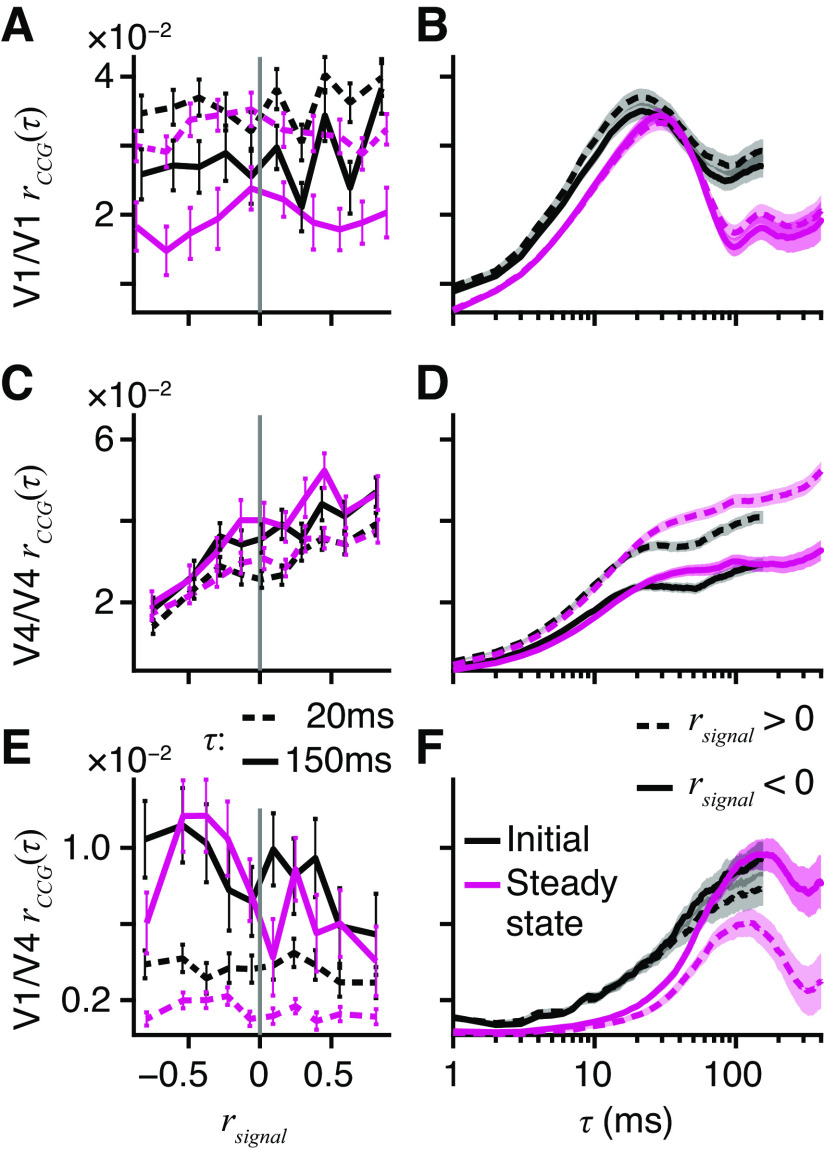
Noise correlations varied with disparity tuning, timescale, and time. *A*: noise correlation (*r*_CCG_) for primary visual cortex V1/V1 as function of signal correlation (*r*_signal_), grouped by timescale (τ) of *r*_CCG_ (20 ms, dashed; 150 ms, solid) and analysis window (Initial, black; Steady state, magenta); each bin has 10% of the data; error bars show SE. *B*: average V1/V1 *r*_CCG_ over timescale τ, grouped by signal correlation (positive *r*_signal_, dashed; negative *r*_signal_, solid) and analysis window, as in *A*; shading shows SE. *C* and *D*: same as *A* and *B* but for extrastriate area V4/V4 pairs. *E* and *F*: same as *A* and *B* but for V1/V4 pairs. spk, Spikes; V1, primary visual cortex; V4, extrastriate area.

We asked initially whether the noise correlations of these neurons had any consistent relationship to disparity selectivity (Fig. 5). We plotted *r*_CCG_ as a function of the signal correlation between pairs (*r*_signal_, *Eq. 9*), grouped by temporal scale (τ; Fig. 5, *A*, *C*, and *E*), or as a function of τ, grouped by *r*_signal_ (Fig. 5, *B*, *D*, and *F*).

For pairs in V4, there was a clear positive relationship of *r*_CCG_ and *r*_signal_ (Fig. 5*C*) at both short (τ = 20 ms, dashed line) and long (τ = 150 ms, solid line) timescales in the Initial (black) and Steady state (magenta) windows (see Table 2). This coincided with an increase in *r*_CCG_ across a range of values of τ for pairs with similar disparity preferences (Fig. 5*D*, *r*_signal_ > 0, dashed line) compared to those with opposite preferences (Fig. 5*D*, *r*_signal_ < 0, solid line). For pairs in V1, a similar but weaker positive relationship was observed (Fig. 5, *A* and *B*) that was significant in the Initial window and the Steady state window for long (τ = 150 ms) but not short (τ = 20 ms) timescales (see Table 2). In summary, neurons in the same cortical area that have similar preferences for the binocular depth of the stimulus tend also to show stronger, more positive noise correlations, suggestive of a local network of neurons that process binocular depth.

**Table 2.** Relationship between r_signal_ and r_CCG_ (accompanies Fig. 5)

Cortical Areas	τ, ms	Initial		Steady State	
		Spearman *r*	*P* value	Spearman *r*	*P* value
V1/V1	20	0.042	0.038*	0.030	0.144, ns
	150	0.050	0.013*	0.054	0.009**
V4/V4	20	0.172	4.133e−16***	0.170	5.674e−16***
	150	0.141	2.448e−11***	0.131	5.906e−10***
V1/V4	20	−0.013	0.392, ns	−0.004	0.779, ns
	150	−0.032	0.032*	−0.025	0.091^•^

ns, Not significant; *r*_CCG_, time-based noise correlation; *r*_signal_, signal correlation; V1, primary visual cortex; V4, extrastriate area V4. *P < 0.05, **P < 0.01, ***P < 0.001; ^•^0.1 > *P* > 0.05.

By comparison, the pattern of correlations between V1 and V4 (Fig. 5, *E* and *F*) were therefore different from within-area correlations in two important ways. First, there was no measurable relationship between *r*_CCG_(20 ms) and *r*_signal_. Second, at longer timescales, a weak relationship emerged, but it was negative (see Table 2). That is, V1/V4 pairs tended to have noise correlations that decreased as the signal correlations increased (as in Fig. 3, *D*–*F*), unlike the V1/V1 and V4/V4 pairs from within the same area (e.g., Fig. 3, *A*–*C*). The pattern of correlations between V1 and V4 suggests that a different principle governs their relationships. These between-area correlations are also more evident at longer timescales.

A concern might be that some of these relationships could be driven by the fact that a dynamic RDS stimulus with the same properties will vary subtly from trial to trial in the exact positioning and sequencing of the dots. Such variation could conceivably induce a local correlation in firing due to similar responses to the details of the dot sequence rather than the same binocular depth. One way to address this is to construct pseudorandom sequences of dot presentation that are in fact identical. This is achieved by creating so-called “frozen noise,” in which the same seed is given to the pseudorandom number generator on every trial, thereby creating identical sequences of dots on repeated trials. We performed some control experiments using frozen noise, comparing neuronal responses with and without resetting of the number seed. These experiments were performed with 100% binocular correlation, as this condition may be expected to give the strongest form of false association between binocular depth sensitivity and dot sequence. As with previous uses of this approach (40), we found that the details of the dot sequence did not matter substantially. Specifically, the information about binocular depth was not artificially inflated when frozen noise, rather than new random sequences, was used to generate the RDS stimuli. That is, using identical dot sequences on each trial did not reduce harmful, information-limiting noise correlations.

### Empirical Evidence of Differential Noise Correlations

A property of information-limiting correlations is that they limit discrimination thresholds in the neighborhood of a specific stimulus value (11). For this reason, they are also called differential correlations, since the concern is with the component of noise that lies along the tuning curve contour corresponding to the stimulus change. Theory (*Eq. 4* in Ref. 11) therefore predicts that measured noise correlations (Σ) are the sum of two sources, one that limits the sensory threshold due to differential correlations (*εf′f′^T^*) and one that does not (Σ_0_):

$$\text{Σ}={\text{Σ}}_{0}+\varepsilon f\prime f\prime^{T}$$

The differential correlations that limit information about the sensory discrimination scale (ε) with the product of the first derivatives (*f*′*f*′) of the two tuning curves from the pair of neurons under consideration (e.g., Fig. 3*C*).

It is here that the specifics of our experimental design present an advantage, because every pair of neurons was tested across a range of binocular disparities, as the psychophysical task was being actively performed. Hence, the relationship between the size of the noise correlation and the value of the derivatives of the tuning curves could be measured at multiple points along the tuning curve for every neuron pair. Moreover, all of these measurements could be directly related to the behavioral performance of the task.

The other component of noise correlations (Σ_0_) does not limit information because, in principle, this source of noise could be eliminated by averaging or pooling across many neurons. Nonetheless, this source of noise does present a practical limitation because it may mask the differential correlations that need to be measured and characterized. We reasoned that the low-variance measure of noise correlation, *r*_CCG_, would be more likely to reveal weak and rapid differential correlations than *r*_SC_, which is noisier. Thus, we examined the relationship of *r*_CCG_ at different integration times (τ) and the product of derivatives over a range of timescales.

**Figure 6.**
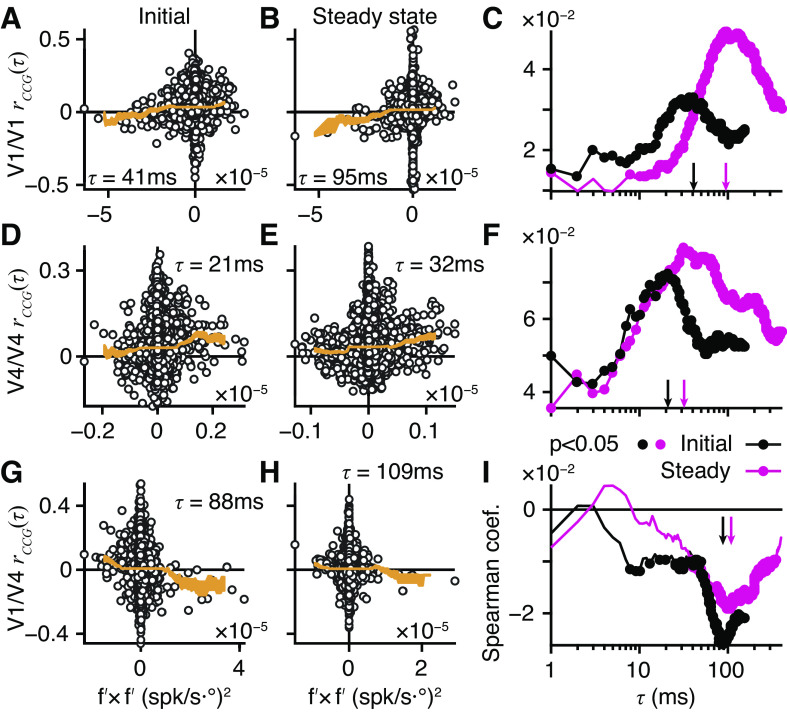
Empirical evidence of differential noise correlations. *A*: *r*_CCG_ as function of temporal scale τ [*r*_CCG_(τ)] from primary visual cortex (V1) at τ = 41 ms plotted over product of first derivatives of disparity tuning curves (*f*′*×f*′), for Initial response phase; *N* = 21,771 cases (2,419 V1/V1 pairs at 9 disparities −0.1° to +0.1°). Running average *r*_CCG_ in orange using window 25% width the range of associated *f*′*× f*′ values at 100 even steps, at center of window. *B*: as *A* but for Steady state; τ = 95 ms. *C*: Spearman correlation coefficient of *r*_CCG_ and *f*′*×f*′ for Initial (black) and Steady state (magenta) responses, at all τ ≤ 400 ms. Filled circles show *P* < 0.05 (2-tailed). Arrows show τ used in *A* (black) and *B* (magenta). *D–F*: same as *A–C* but for extrastriate area V4/V4 pairs and using τ = 21 ms for *D* and τ = 32 ms for *E*, with *N* = 20,034. *G–I*: same as *A–C* but for V1/V4 pairs and using τ = 88 ms for *G* and τ = 109 ms for *H*, with *N* = 41,382. *r*_CCG_, Time-based noise correlation.

For pairs of neurons from the same area (V1 or V4), noise correlations had a positive relationship with the product of derivatives (*f*′* × f*′; see *Eq. 23*) during both the Initial and Steady state phases of neuronal activity (Fig. 6). This relationship is shown for some specific values of timescale (τ), plotting *r*_CCG_ against *f*′* × f*′ (V1, Fig. 6, *A* and *B*; V4, Fig. 6, *D* and *E*; running averages in orange). A continuous plot of the slope (Spearman correlation) between *r*_CCG_ and *f*′* × f*′ across timescales (τ) is shown in Fig. 6*C* for V1 and in Fig. 6*F* for V4; these continuous plots were used to select timescales for the illustrative plots (Fig. 6, *A*, *B*, *D*, and *E*). The vertical arrows on the timescale axes show the τ values where the data peak for V1 (Fig. 6*C*) and V4 (Fig. 6*F*). Statistical summaries for the plots in Fig. 6, *A*, *B*, *D*, and *E*, are shown in Table 3. It is evident that the relationship between *r*_CCG_ and *f*′* × f*′ is most prominent in a narrow range of timescales (10 ⪅ τ ⪅ 100 ms).

**Table 3.** Summary of relationship between r_CCG_ and f′×f′ (accompanies Fig. 6)

Cortical Areas	Initial			Steady State		
	τ, ms	Spearman *r*	*P* value	τ, ms	Spearman *r*	*P* value
V1/V1	41	0.033	1.137e−6***	95	0.049	3.423e−13***
V4/V4	21	0.072	1.437e−24***	32	0.079	2.726e−29***
V1/V4	88	−0.026	1.023e−7***	109	−0.019	9.298e−5***

*r*_CCG_, Time-based noise correlation; V1, primary visual cortex; V4, extrastriate visual area V4. ***P < 0.001.

For pairs of neurons recorded simultaneously between V1 and V4, the result was unexpected (Fig. 6, *G*–*I*). Instead of a positive relationship, we observed a consistent negative relationship of *r*_CCG_ and *f*′* × f*′ in the Initial and Steady state time periods, which peaked for a narrow range of τ close to 100 ms (Fig. 6, *G*–*I*). This negative relationship of *r*_CCG_ and *f*′* × f*′ does not mean that the values of *r*_CCG_ are themselves negative. On average, the values of *r*_CCG_ and indeed of *r*_SC_ are positive (see Fig. 4). It is how these positive values of *r*_CCG_ vary with the size and sign of *f*′* × f*′ that is of concern here, similar to the between-area relationship of *r*_signal_ and *r*_CCG_, mentioned above (Fig. 5 and Table 2).

We confirmed these results by performing a series of ANCOVA tests to remove factors that might have led *r*_CCG_ to falsely appear correlated with *f*′* × f*′. Explanatory factors were introduced in the following order with type I sum of squares: experimental day, analysis window (Initial and Steady state), τ (20 ms and 100 ms), geometric mean firing rate (from measured tuning curves), and *f*′* × f*′. The geometric mean and *f*′* × f*′ were continuous predictors, whereas the others were categorical. Thus, day-to-day variation as well as modulations in firing rate due to different disparity conditions were both accounted for. The key parameter of *f*′* × f*′ remained a strongly significant predictor of *r*_CCG_ in all cases (*P* < 0.002). This result held when the analysis was repeated using only the example sessions.

### Differential Noise Correlations Are Attenuated at Long Timescales

The amount of information that can be encoded by a neuronal pool depends fundamentally on the number of different firing states that the pool can adopt. Thus, correlation among members of the pool reduces the capacity for information coding. For a sensory discrimination task, the impact of these correlations depends on the discrimination task that is to be performed. Differential correlations that mimic the change in neuronal response to the stimulus change that must be identified will result in a saturation of the information in a neuronal pool of a certain size. That is, differential correlations will follow the same principle identified by earlier studies, which concluded that threshold performance as a function of pool size is limited by correlation in the neuronal spike counts (6, 7).

**Figure 7.**
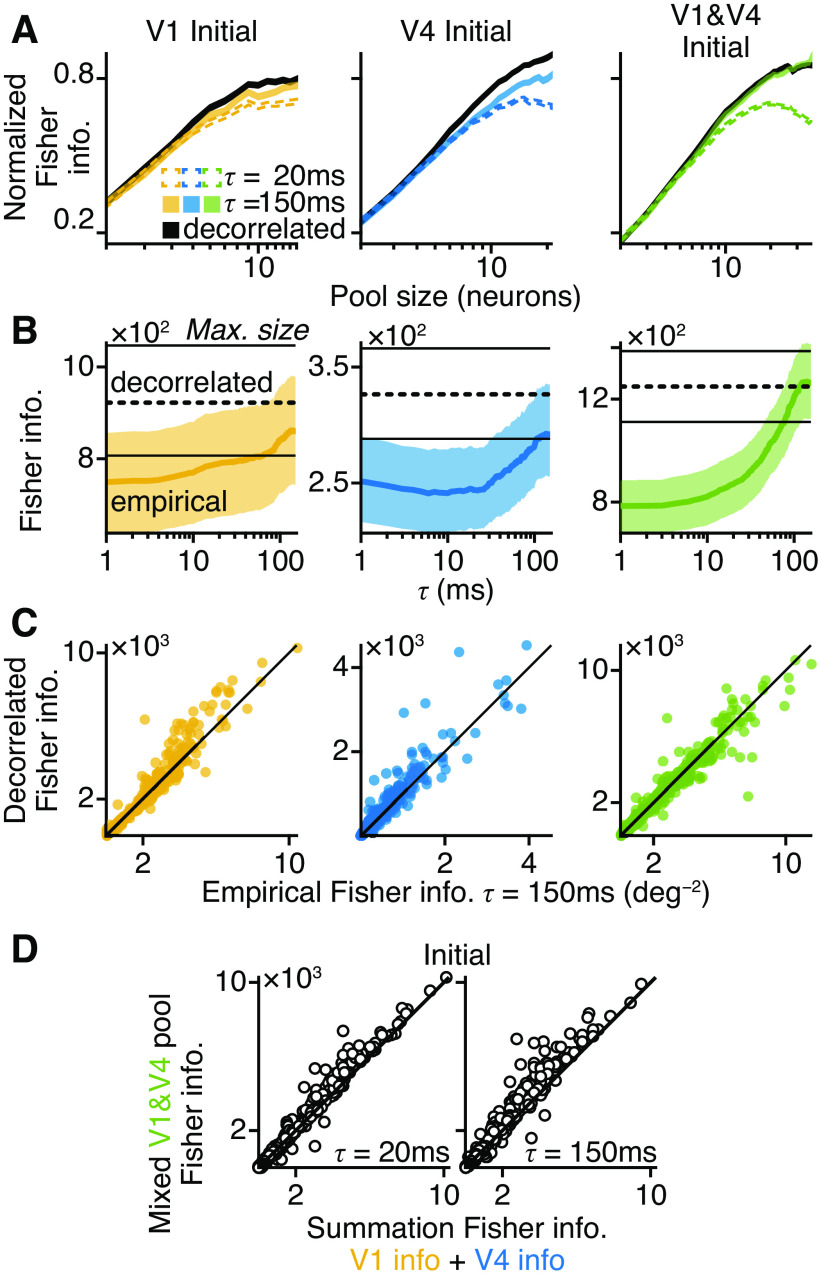
Differential noise correlations are attenuated at long timescales. *A*: average ±SE normalized bias-corrected linear Fisher information, over neuronal pool size. Information shown for rapid (τ = 20 ms, dotted), slow (τ = 150 ms, solid), and no (black) noise correlations; *N* = 780 (78 disparity pairs × 10 experiments). Units added to pools by decreasing order of Initial disparity mutual information (DMI). *B*: average Fisher information across *r*_CCG_ τ. Information is empirical (thick solid) or decorrelated (dashed). 95% bootstrap confidence interval (CI). *C*: decorrelated vs. empirical information at τ = 150 ms. *D*: information of mixed primary visual cortex (V1)/extrastriate area V4 pool (size 12) vs. summed information across V1-only and V4-only pools (size 6) for rapid (*left*) and slow (*right*) correlations. Max. pool sizes in *B* and *C*. For *B–D*, units of Fisher information are degrees^−2^. *r*_CCG_, Time-based noise correlation.

Therefore, we measured the information about binocular depth that was available within neuronal pools of increasing size (Fig. 7*A*). Information was assessed as linear Fisher information (*Eq. 24*; Ref. 12), which measures how accurately the responses of the neurons in a pool can determine which binocular depth was presented. Fisher information is inversely related to the width of the likelihood function for estimating binocular depth. It therefore increases when the population response can better distinguish between different RDS depth values, and an increase of Fisher information is brought about from the addition of informative neurons or from changes in the pool’s correlations (14). Empirically, information-limiting correlations can be removed from measured samples of neuronal population activity by shuffling the relationship between neuronal activity across multiple presentations. Shuffling decorrelates the population activity and is therefore predicted to increase the Fisher information, if information-limiting correlations are present in the experimental measurements. Here (Fig. 7), we use Fisher information only as an analytical tool to quantify the amount of disparity information in different groupings of simultaneously recorded neural signals. Below, we explore how V1 and V4 signals might be combined at the level of V4 (see Fig. 11).

Using responses from the Initial phase of firing, we calculated the normalized and averaged bias-corrected linear Fisher information (*Eq. 30*) for pools of units from V1 only (Fig. 7*A*, *left*, gold), V4 only (Fig. 7*A*, *center*, blue), and V1 mixed with V4 (Fig. 7*A*, *right*, green). Units were added to each pool from the most selective for disparity down to the least selective (45); thus, part of the apparent saturation is due to exhaustion of informative units rather than the effect of correlations. The range of pool sizes was limited to those observed in at least half of the experimental sessions. The Fisher information available from experimental recordings was compared against the information within an equivalent pool of decorrelated units (Fig. 7*A*, solid black curve), for the cases of rapid (τ = 20 ms, Fig. 7*A*, dotted curve) and slow (τ = 150 ms, Fig. 7*A*, solid colored curve) noise correlations. For all pool types, we found that the empirical information at τ = 20 ms began to diverge from the decorrelated information for pools of 10 units. In pools with only V1 or V4 units, there was a similar but weaker divergence when τ = 150 ms. However, for the mixed V1 and V4 pool, the empirical information at τ = 150 ms was closely aligned with the decorrelated information (Fig. 7*A*, *right*).

To get more detail about the effect of timescale, we plotted the average, raw (not normalized) Fisher information of the Initial responses as a function of τ (Fig. 7*B*), using the maximum available pool size for each pool type in each experimental session. The empirical information (Fig. 7*B*, solid colored line) was compared against the decorrelated information (Fig. 7*B*, dashed black horizontal line). For all pool types, the empirical information tended to increase with τ, but the empirical information converged on the decorrelated information only in the mixed V1 and V4 pool, at around τ = 100 ms.

We confirmed this result by plotting the Fisher information after decorrelation against the empirical information for τ = 150 ms (Fig. 7*C*). The information after decorrelation was greater than empirical for the single area pools [V1: mean paired difference (PD) = 66.117°^−2^, paired-sample *t* test *P* = 4.700e−6; V4: PD = 32.316°^−2^, *P* = 1.745e−7), but there was no significant difference for the mixed pool (V1/V4: PD= −20.558°^−2^, *P* = 0.183). Similar results were observed using Steady state responses (not shown), except that the empirical information never entirely converged on the decorrelated information over the range of timescales that we analyzed.

The PD results were verified with our example recording sessions at the standard values of τ. For V1- or V4-only pools all available units were used, but the mixed population contained only V4 units with RFs close to V1’s (<7.99°). As an additional control measure, disparities were limited to a range of fine values (−0.05° to +0.05° inclusive, 21 pairings) to more effectively compare population responses to only small deviations of the stimulus value. However, our result remained robust at the single-session level, showing that PD was significantly greater than zero in all conditions (*P* ≤ 3.289e−3), except for the mixed V1 and close V4 population in the Initial window (*P* ≥ 0.717).

In another control, we tested whether the increase in the mixed V1/V4 information might be due to its larger pool size, compared to the V1- or V4-only pools, rather than any differences in the structure of the noise correlations. This was tested by plotting the information of a mixed pool with 12 units against the sum of information from separate V1- and V4-only pools of size 6 each (e.g., Initial information shown in Fig. 7*D*); as in Fig. 7*A*, the most informative units were used. The average paired difference in information revealed that the mixed pool had extra information, over a range of values for τ in both the Initial and Steady state windows (paired *t* test, *P* < 0.05). This difference increased with τ; hence information increased in the mixed pool as its covariance structure changed.

### Differential Correlations and Receptive Field Separation

A proper analysis of this data set requires consideration of the relative positions of the receptive fields (RFs) recorded from V1 and V4. Because of the topographic placement of the chronic Utah arrays, the majority of V4 RFs were more eccentric than the V1 RFs. Furthermore, the average distance between V1 and V4 RFs was greater in one monkey (*M135*) than the other (*M138*). During our main experiments, we presented the neurons from areas V1 and V4 with a single visual stimulus that extended across the RFs of both sets of neurons from these visual areas. Both set of RFs were therefore coactivated by a single RDS stimulus (Fig. 2*A*).

Nonetheless, it could be argued that the presence of a correlation structure that is the opposite of normal information-limiting correlations is simply due to the RF separation rather than the fact that the recorded neurons were in different cortical areas. Furthermore, the apparent cancellation of information-limiting noise might have been caused by the misalignment of V1 and V4 RFs rather than any genuine corrective mechanism. To address these concerns, we can formulate two testable null hypotheses. First, when compared over a range of similar values of RF separation, there will be no difference in the structure of within-area and between-area correlations. Second, any measurable recovery of information by a population of V1 and V4 neurons should increase as the separation of V1 and V4 RFs increases.

Information-limiting noise correlations produce a positive relationship between measured noise correlations and the product of tuning curve derivatives (*f*′* × f*′); for brevity, we refer to this relationship as R_D_. Our main analysis revealed a positive R_D_ between pairs of neurons from the same cortical area, providing direct evidence of information-limiting noise correlations within both V1 and V4 for encoding binocular disparity. However, pairs of neurons from different cortical areas had, on average, a negative R_D_.

**Figure 8.**
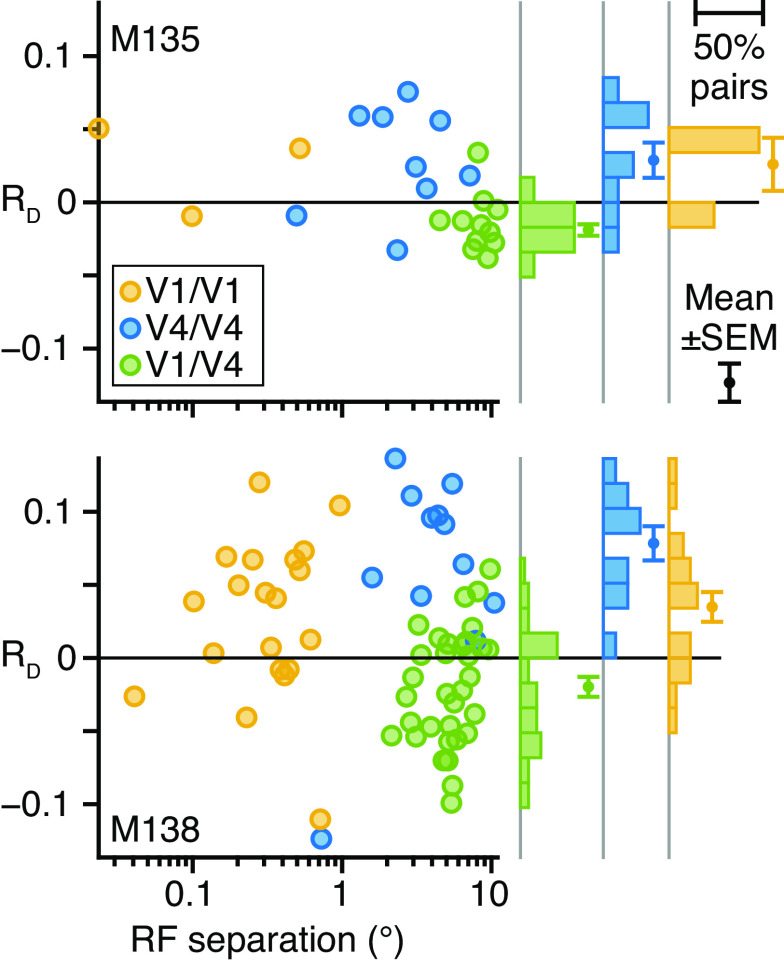
Differential correlations and receptive field (RF) separation. For each type of neuron pair, pairs were ordered by RF separation and then grouped; at least 100 pairs to each group (*N* bins = *N* pairs/100, rounded down). R_D_ [Spearman correlation of *r*_CCG_(150 ms) vs. *f*′*×f*′] for the pairs in each group as a function of average log_10_ RF separation. Marginal histograms show distribution of R_D_, counting groups. *r*_CCG_, Time-based noise correlation.

Plotting R_D_ as a function of RF separation in Fig. 8 shows that within-area correlations are generally positive, on average, whereas correlations between areas are predominantly negative—across the spatial separations in our sample. Neuron pairs were first binned (1 circle/bin) by their RF separation, with at least 100 pairs/bin [*N* bins; *M135* V1/V1 = 3, V4/V4 = 9, V1/V4 = 10 (with outlier 11); *M138* V1/V1 = 19 (20), V4/V4 = 11 (12), V1/V4 = 33]; binning was done after discarding the 2% of pairs with the lowest DMI, i.e., the least disparity selectivity. The R_D_ of each bin was quantified as the Spearman correlation of measured noise correlations [*r*_CCG_(150 ms)] and *f*′* × f*′ of the bin’s pairs. Likewise, the average RF separation was found for each bin. There was no provable relationship between RF separation and differential correlations. If anything, our data indicate that all relationships, positive and negative, declined as the spatial separation of RF centers increased.

To emphasize this point, consider that previous control analyses of V1/V4 R_D_ in this study used pairs with RFs that were close to each other (<7.99°). If we now do the opposite and constrain ourselves to pairs of neurons with distant RFs (>7.99°), then the R_D_ of V1/V4 pairs at τ = 100 ms is no longer significantly different from zero (*M135* Spearman R_D_ = 0.018, *P* = 0.124; *M138* R_D_ = 0.021, *P* = 0.208).

In summary, our data support two conclusions. First, there is a categorical difference between within-area correlations and between-area correlations, regardless of RF separation. Second, information recovery between V1 and V4 is stronger when RF separation is smaller, not when it is larger.

### Differential Correlations and Behavioral Choice

In the final phase of the trial (Fig. 1*Aiv*), the binocular depth instantaneously changed in one of the four RDS stimuli. This popout event revealed one out of the four RDS stimuli to be an oddball target. The monkey could get a reward by correctly choosing the target RDS with a saccade onto that stimulus. Rewards were given after the gaze fell reliably onto the target RDS (correct choice). Given that we recorded neuronal responses to just one of these four RDS locations, we divided the trials into four categories of outcome, specified by combinations of two factors: Target IN or OUT of the RF and CORRECT or INCORRECT choice. Trials were only used in the behavioral analyses if 117 ms ≤ RT ≤ 344 ms for *M135* and 143 ms ≤ RT ≤ 463 ms for *M138*; this maximized the likelihood that the subjects made informed choices based on the RDS stimuli, as opposed to guessing (see Fig. 1*C*).

**Figure 9.**
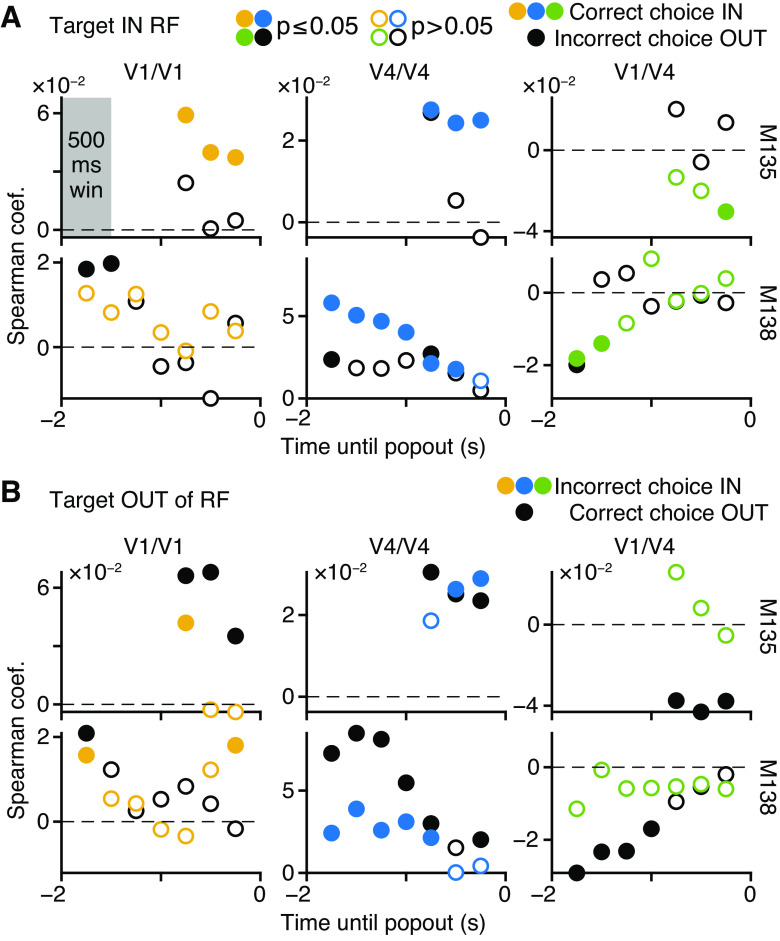
Effect of choice on direct evidence of differential correlation. Spearman correlation of *r*_CCG_(τ) vs. *f*′*×f*′ (see Fig. 6) in 500-ms window (gray) at 250-ms steps (circles, centered in each window) prior to popout event (Fig. 1*Aiv*). For primary visual cortex (V1)/V1 (*left*, τ = 30 ms), extrastriate area V4/V4 (*center*, τ = 30 ms), and V1/V4 (*right*, τ = 110 ms) pairs. Random dot stereogram (RDS) onset (Fig. 1*Aiii*) at −1 for *M135*, −2 for *M138*. Circle fill shows significance (2-tailed, *P* ≤ 0.05). Colored circles show choices IN to receptive field (RF); black circles show choices OUT of RF. *A*: popout target IN the RF; choices IN are correct. *B*: popout appears OUT of RF; choices IN are incorrect; incorrect choices OUT of RF not shown. *r*_CCG_, Time-based noise correlation.

The association between behavioral choice and the neuronal state is plotted in Fig. 9. The *x*-axis shows time relative to the popout event. As before, the state of the noise correlation structure of the recorded neuronal responses, R_D_, is summarized by the Spearman correlation between *r*_CCG_(τ) and the product of differentials *f*′* × f*′. Values of τ were 30 ms for the V1/V1 and V4/V4 pairs and 110 ms for the V1/V4 pairs, which deviates from our standard set for illustrative purposes (but see ANOVA below). Owing to differences between animals that emerged in early training, the two monkeys were exposed to either 1 s (*M135*) or 2 s (*M138*) of indistinguishable RDS stimuli during the Presentation phase (Fig. 1*Aiii*), before the popout event. In a fraction of catch trials, no depth change occurred, to measure guess rates; these trials were discarded from this analysis.

The data in Fig. 9 show an association between the correctness of choice and the state of correlations, R_D_, before the behavioral decision. The effect is strongest for the within-area correlations of V4 (Fig. 9*A*, *center*, blue/black data points, fill shows 2-tailed significance at 5% level). For both animals, correct choices onto the target over the RF are associated with stronger values of R_D_ in V4. This is not due to an alteration of correlations by the target stimulus, because the target is still unknown. Second, analysis of the opposite contingency (Fig. 9*B*), when the target occurs outside of the RF, shows that these correlations are also stronger for correct choices. In this case, a correct choice means selecting a target outside of the RF and choosing the target over the RF is incorrect. For cases where both the target and incorrect choices were out of the RF, the R_D_ measure of differential correlation was similar to when incorrect choices went into the RF (not shown). Therefore, this association in V4 is between correctness of choice and the measured state of differential correlations, regardless of target location. For the within-area correlations of V1 (Fig. 9, *left*, gold/black data points), the same general trend is present, but the effects are much weaker and less consistent, especially in monkey *M138*.

For the between-area correlations of V1/V4 (Fig. 9, *right*, green/black data points), an interesting pattern emerges. Previous analyses, above in Figs. 6 and 8, demonstrated a negative Spearman correlation for the V1/V4 R_D_. This negative relationship is even stronger for correct choices as opposed to incorrect choices, most notably when the target is outside the RF (but note that target IN trial count is ≈25% of target OUT).

To test the significance of these changes, an all-ways ANOVA was performed on the normalized ratio of *r*_CCG_ over *f*′* × f*′ (*Eq. 33*), separately per subject, testing the effects of recording day, time (window position), cortical area (V1 only, V4 only, V1/V4), and choice (correct or incorrect). The standard set of noise correlation timescales were used (τ = 20 ms V1/V1 and V4/V4 pairs, τ = 100 ms V1/V4), for a more direct comparison with our other results. The normalization permitted the pooling of target IN and OUT trials, because both sets show a similar dependence on correctness. The effect of choice was significant (except for *M135* V1 only, *P* ≥ 0.159) when controlling for day, time, and cortical area (Tukey–Kramer multiple-comparisons test, *P* < 0.001). These results held even when the data were restricted to the example recording days.

By themselves, these results do not necessarily confirm that differential correlations are stronger in magnitude when correct choices are made (that is, more positive for within-area correlations or more negative for between-area correlations). Another interpretation is that correct performance is associated with a suppression of general, nondifferential correlations within the neuronal pool and that this suppression allows a stronger coupling between information-limiting correlations and task performance.

**Figure 10.**
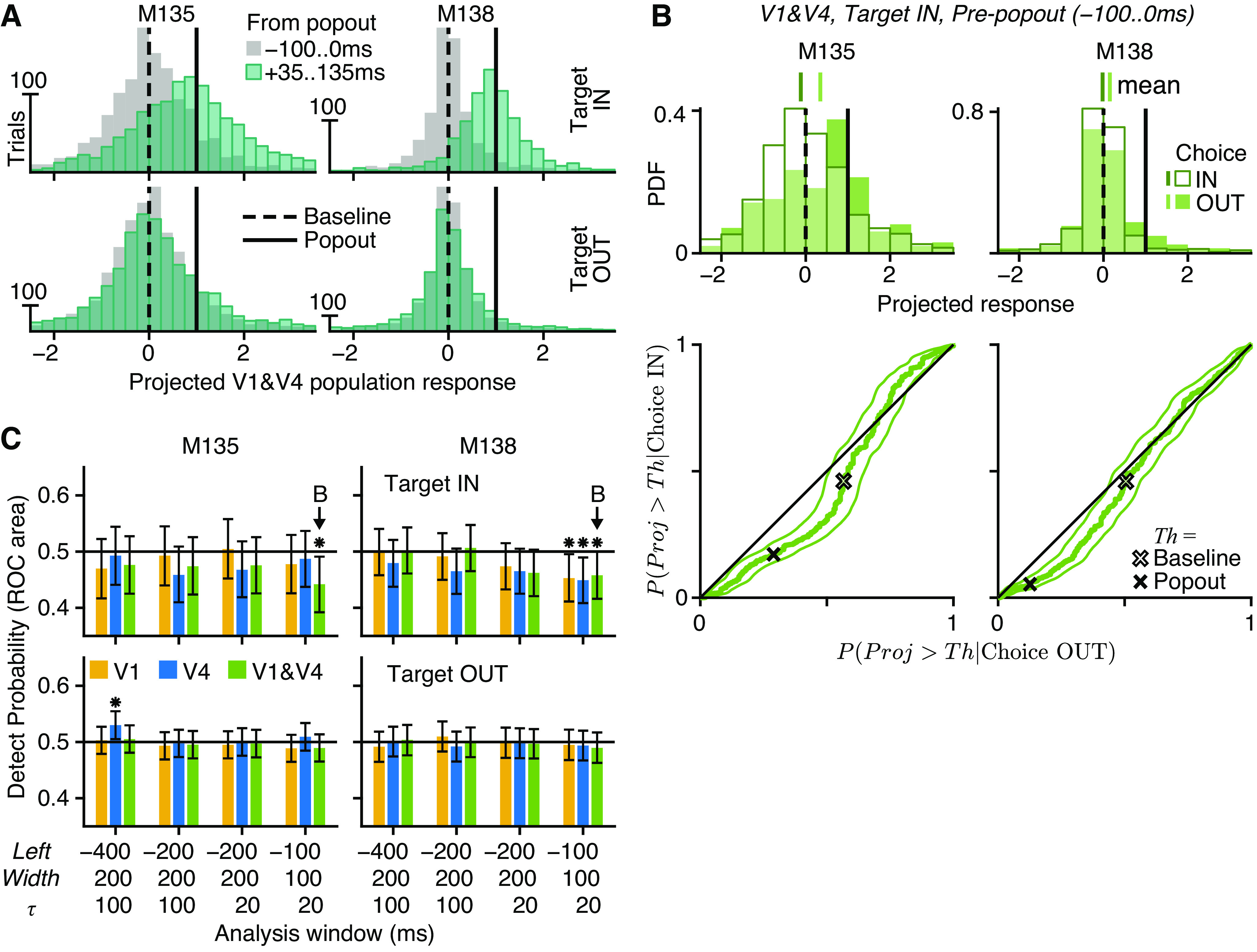
ifferential correlation predicts choice. *N*-dimensional firing rates of simultaneous population responses were linearly projected onto the axis that optimally discriminated baseline from popout responses. *A*: example projected responses for combined primary visual cortex (V1) and extrastriate area V4 populations. Mean value of 0 for response to baseline disparity (dashed line) and +1 for popout (solid line). Target random dot stereogram (RDS) appeared IN (*top*) or OUT (*bottom*) of the receptive field (RF). Responses before (−100 to 0 ms, gray) or after (+35 to +135 ms, teal) start of popout event. *B*, *top*: distributions for each monkey grouped by choice IN (empty bars) or OUT (filled bars) of RF; black vertical lines as in *A*, green vertical lines show averages. *Bottom*: example receiver operating characteristic (ROC) curves (green) using prepopout projections from *A* (*top*, gray); black line, chance association. Crosses show probability values equal to expected baseline (open) or popout (filled) response. *Th* in axis labels refers to the sliding threshold value used to calculate the ROC curves. *C*: detect probability (DP) for populations with V1 units only (gold), V4 units only (blue), or mixed V1 and V4 units (green), relative to chance (0.5, black horizontal line) with target RDS IN (*top*) or OUT (*bottom*) of the RF. Population responses taken from analysis windows at different positions (left edge = −400, −200, or −100 ms before popout) and width (200 or 100 ms). Projection weights optimized for noise correlations at *r*_CCG_ timescale τ = 20 or 100 ms. *95% confidence interval (CI) different from chance. Arrows show DP for curves in *B*. All error bars are 95% bootstrap CI (2e4 boot samples, percentile method). Neuron pairs’ separation from RF (RF_separation_) < 7.99°. PDF, probability density function; *r*_CCG_, time-based noise correlation.

More direct evidence of the potential importance of differential correlations is presented in Fig. 10. Here, the goal is to obtain a unidimensional measure of the differential correlation in a neural population on each trial, so that its trial-to-trial relationship with perceptual choice could be quantified. The focus is upon the change in the V1 and V4 population response that is caused by the popout stimulus. By projecting the population response onto the optimal axis for discriminating baseline versus popout disparity (*Eq. 34*), we reduce the *N*-dimensional firing rate down to a 1-dimensional measure of the entire population response. This is done by taking a weighted, linear sum of the firing rates, using weights given by *w* ∝ Σ^−1^Δ*F*, for population covariance Σ and expected size and sign of response to the popout Δ*F*; weights were optimized according to the baseline and popout disparity on each trial. Owing to the Σ^−1^ term, the optimal axis can mitigate nondifferential noise correlations, but any differential correlations in the population response are necessarily projected onto the optimal axis.

The values arising from this projection were normalized so that the expected response to the baseline disparity was 0, and the expected response to the popout disparity was always +1, regardless of whether it is actually an increase or a decrease in disparity. If prepopout values for the projected response are near 0, this suggests that the population contained very little differential correlation and accurately encoded the baseline disparity. Values between 0 and +1 suggest the presence of differential noise that tilted the population closer to the firing pattern expected in response to the popout disparity, whereas values between −1 and 0 suggest the opposite. Hence the relative direction and magnitude of perturbations in disparity encoding could be estimated on each trial.

Figure 10*A* shows the distributions of projected responses from each animal from the combined population of all V1 units with the V4 units that had RFs close to the V1 RFs (V1 to V4 RF_separation_ < 7.99°). When the analysis window is −100 ms to 0 ms (Fig. 10*A*, gray) before the popout event, then the distributions peak around 0, regardless of the upcoming target location. After the start of the popout event (+35 ms to +135 ms window, Fig. 10*A*, teal), the distributions shift toward +1, for the case when the popout target was IN the RF (Fig. 10*A*, *top*). For the other cases, when the target was OUT of the RF, then the pre- and postpopout projection distributions were indistinguishable (Fig. 10*A*, *bottom*). Thus, the normalized projection succeeded in representing the combination of signal and noise in encoding the RDS disparity.

### Differential Correlations Limit Perceptual Choices

We asked whether perturbations in this projected response that occur just before the arrival of the popout stimulus might affect the ability of the animals to detect the upcoming change in the visual stimulus. We quantified this by computing a version of detect probability (DP; Ref. 41) to measure the covariation of the normalized projection with the perceptual choice. Projected responses from the time period −100 ms to 0 ms were grouped according to behavioral choice [Fig. 10*B*, *top*, Choice IN (open) and OUT (filled) of the RF]. These distributions, categorized by future choice, allow the computation of receiver operating characteristic (ROC) curves (Fig. 10*B*, *bottom*, green), where the choice IN rate is plotted on the vertical axis of the ROC curve and choice OUT is on the horizontal axis. The DP is the area under this ROC curve (*Eq. 38*), such that a DP > 0.5 indicates that larger, more positive perturbations preceded choices IN to the RF, and DP < 0.5 suggests the opposite and DP = 0.5 indicates no effect on detection. Figure 10*B* shows that both ROC curves dip below the chance line (black). This means that positive perturbations that mimic the arrival of the target stimulus and occur just beforehand have a negative effect on detection rates for the target.

It is interesting to note that the portions of the probability distributions of the projected response that are most relevant for this effect on behavior lie exactly within the bounds that are also relevant for the neural detection of the target stimulus. The relevant positions are marked as the baseline (empty cross) and popout (filled cross) disparities and are equivalent to the vertical lines in Fig. 10*A*. The perturbations in the prepopout period have their greatest impact on behavior, producing a DP < 0.5, when they occur within these boundaries.

In Fig. 10*C*, we computed DP for populations of units, with RF_separation_ < 7.99°, for V1-only (gold), V4-only (blue), or mixed V1 and V4 (green) units, using responses measured from a range of prepopout windows (−400 to −200, −200 to 0, −100 to 0 ms), with weights optimized with either fast (τ = 20 ms) or slow (τ = 100 ms) noise correlations; values were computed separately for trials with the target IN the RF (Fig. 10*C*, *top*) or OUT (Fig. 10*C*, *bottom*). We found that the only difference in DP from 0.5 that was consistent across subjects was in the combined V1 and V4 responses just prior (−100 to 0 ms) to the appearance of the popout target IN the RF (Fig. 10*C*, arrows). When the same analysis was restricted to the example recording days, then no difference in DP from 0.5 was found, but the relatively small number of trials may not have had sufficient statistical power for the nonparametric method used here. Notably, for the pooled data, the relevant temporal scale for these noise correlations was fast, at 20 ms. This suggests that rapid, prepopout differential correlations that spanned both V1 and V4 are detrimental to detecting the change in disparity.

This is a key prediction of the role for differential correlations in limiting the detection of the stimulus. When the state of the network before the arrival of popout is such that it more closely resembles the state of the network after the popout stimulus arrives, then this is detrimental to perception of the disparity change. On trials when the fluctuations in network state place it further away from the neural response to the popout, then detection of the popout target is less impaired. In line with Figs. 6 and 7, our results suggest that it is the rapid differential correlations that do indeed degrade behavioral detection. Slower correlations before the popout event do not appear to impair detection, suggesting that integrative processes in the visual cortex may have enough time to attenuate rapid differential fluctuations.

### A Model V4 Output Neuron Can Attenuate Differential Noise

Here we consider how the joint actions of V1 and V4 could preserve information transmission in the presence of information-limiting correlations within each individual cortical area. The finding of pairs of V1 and V4 units that had opposite disparity preferences and positive noise correlations (e.g., Fig. 3, *D*–*F*) provides evidence for one route to canceling the correlated noise without reducing the sensory signal (8, 10). This phenomenon has been observed experimentally in the second-order areas of the somatosensory system (46).

**Figure 11.**
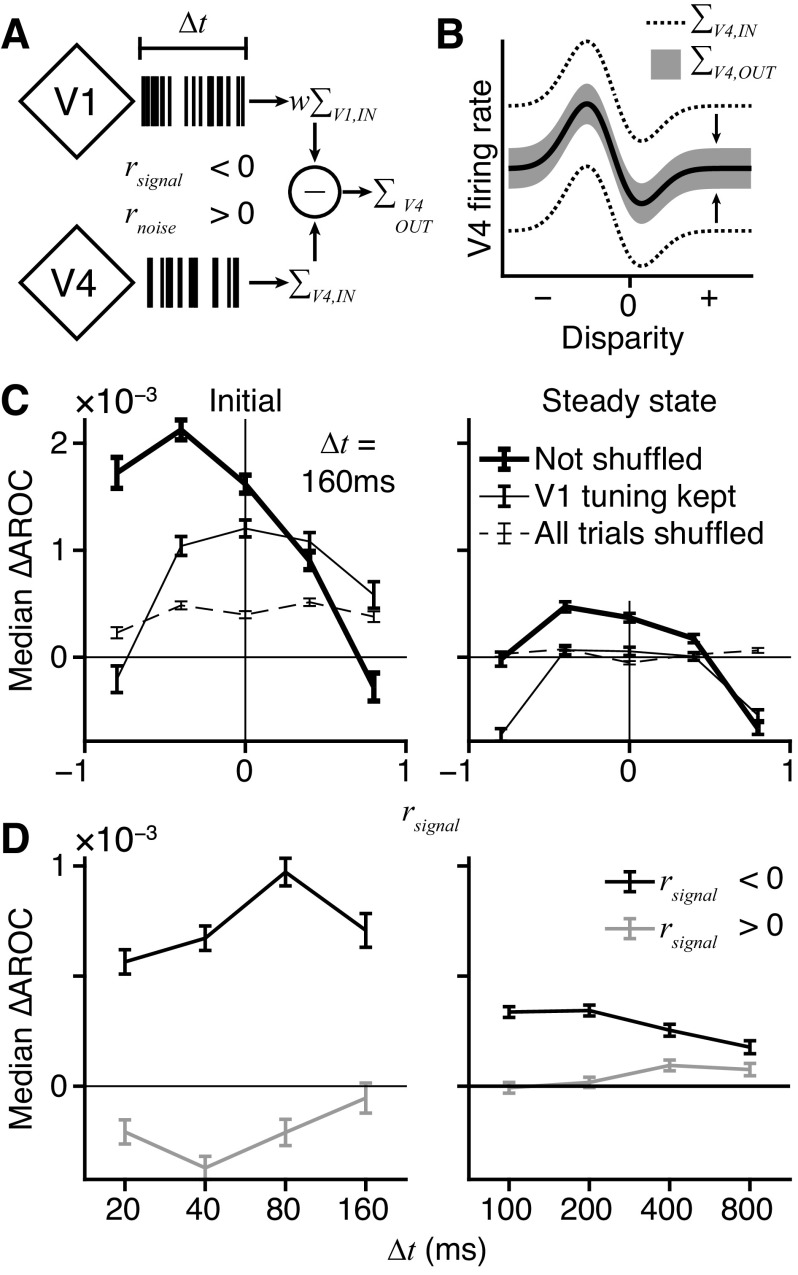
A model extrastriate area V4 output neuron attenuates differential noise. *A*: diagram of model V4 output neuron. Diamonds, afferent neurons. Rasters, afferent spikes in Δ*t*-ms window. Weighted primary visual cortex (V1) count (*w*Σ_V1,IN_) subtracted from V4 count (Σ_V4,IN_) gets V4 output count (Σ_V4,OUT_). *B*: disparity tuning prediction, afferent V1/V4 signal correlation (*r*_signal_) < 0 and noise correlation (*r*_noise_) > 0. V4 input variance (Σ_V4,IN_, dotted lines). Model V4 output variance (Σ_V4,OUT_, gray shading). *C*: average change in area under the receiver operating characteristic curve (AROC) (V4 output vs. V4 input) across *r*_signal_ of afferent V1/V4 pairs, Δ*t *=* *160 ms. V1 responses shuffled across trials to break V1/V4 correlations but not disparity selectivity (V1 tuning kept, thin line), to break correlations and V1 tuning (All trials, dashed line), or not shuffled (solid line). *N* = 358,644 (78 baseline disparity pairs × V1/V4 pairs). *D*: average difference in AROC over Δ*t*, V4 model output AROC with no shuffling minus shuffled AROC with preserved V1 tuning. Afferent V1/V4 *r*_signal_ < −0.5 (black) and *r*_signal_ > +0.5 (gray). *C* and *D*: initial data on *right*; Steady state on left. Error bars show SE. *N* = 4,598 model V4 output neurons, 1 per recorded V1/V4 pair.

To investigate this further, we built a simple model of a V4 output neuron (Fig. 11; *Eqs. 35–37*), of a type that might project to downstream areas. The model had two afferent neurons (Fig. 11*A*), a neuron that projects from V1 onto the V4 output neuron and an intrinsic, local-circuit neuron from within V4 that also connects to the V4 output neuron. The details of this could be varied without a loss of generality, but importantly this represents a simple feedforward system that can explain many of our results.

Correlated noise was removed by subtracting the weighted V1 responses from the input V4 responses, each input integrated across a Δ*t*-ms window, to produce the V4 output responses. Each V1 and V4 pair in our data set was used to simulate the responses of a model V4 output neuron, whose disparity selectivity was then measured. The prediction is that the V1/V4 input pairs with positive noise correlation (*r*_noise_ > 0) and negative signal correlation (*r_signal_* < 0) should yield V4 output neurons with better disparity selectivity (Fig. 11*B*; dashed line, V4 input variance; gray, output V4 variance).

We test this circuitry directly with empirical data by measuring the disparity selectivity of each simulated V4 unit, using the area under the receiver operating characteristic curve (AROC, *Eq. 38*) as a measure of neuronal sensitivity. Focusing on the Initial analysis window, the difference in AROC (*Eq. 39*) between the V4 model output and input (Fig. 11*C*, *left*, thick solid line, Not shuffled) confirmed the prediction above, where positive differences indicate model V4 output neurons with better disparity sensitivity.

As stated, a pair could improve its stimulus sensitivity either by changing its correlation structure or by producing a larger modulation of firing in response to stimulus changes. Hence, it is possible that the superior performance of the model V4 output neuron was trivially due to the combined signaling of the V1 and V4 input neurons, rather than any cancellation in correlated noise. We tested this by recomputing the model V4 outputs after shuffling the afferent V1 responses between trials such that the V1 unit maintained its stimulus sensitivity but the noise correlations were destroyed (Fig. 11*C*, *left*, thin solid, V1 tuning kept). In this scenario, the model V4 neuron can only improve its sensitivity through combined input signals but not through canceling out the correlated noise of its afferents.

Model V4 neurons had better stimulus sensitivity than the afferent V4 neuron, whether there was shuffling (Fig. 11*C*, *left*, thin solid line) or not (thick solid line). Yet, the model V4 neurons with no shuffling achieved a further increase in stimulus sensitivity in comparison to those with shuffling. This improvement in sensitivity occurred specifically when the afferent neurons had opposite disparity selectivity (*r*_signal_ < −0.5, mean difference AROC = 2.961e−3, paired *t* test *P* = 4.974e−112); in contrast, the model without shuffling suffered a reduction in sensitivity when the afferent neurons had similar disparity selectivity (*r*_signal_ > +0.5, −7.894e−4, *P* = 3.269e−10). This is precisely the pattern that should be observed when the afferent V1 and V4 neurons have positive noise correlations (8, 10). Additional stimulus sensitivity is available by canceling out the noise correlations of afferent neurons with opposite stimulus selectivity.

As differential correlations seemed to be reduced by slower, V1 to V4 correlations (Fig. 7), we asked whether the simulated V4 output neuron would improve its performance with a wider integration window. Thus, we evaluated the Initial V4 output responses over a set of Δ*t* values. For each Δ*t*, the unshuffled model V4 sensitivity (Fig. 11*D*, *left*) was subtracted by the shuffled model V4 sensitivity; again, this tests for changes in sensitivity due to the cancellation of afferent noise correlations. When the input V1/V4 pair had *r*_signal_ < −0.5, there was a significant tendency for sensitivity to increase as Δ*t* increases (Δ*t* = 20 ms vs. 160 ms, mean paired difference = 1.540e−3, *P* = 9.490e−25).

For the Steady state responses, the AROC measure detected a weak improvement (Fig. 11*C*, *right*) when the input pair had opposite disparity preferences (Not shuffled vs. V1 tuning kept, mean paired difference = 4.9181e−4, paired *t* test *P* = 3.1995e−26). No improvement in selectivity was found with increasing Δ*t* (Fig. 11*D*, *right*, Δ*t* = 100 ms vs. 800 ms, mean paired difference = −3.902e−4, *P* = 7.986e−16).

## DISCUSSION

Cortical areas V1 and V4 contain noise correlations that could limit the information that those areas convey about stereo depth. This noise limits information chiefly at short timescales, as the animal is discriminating depth. Unexpectedly, noise correlations between the two areas were found to have a different structure at longer timescales, which could reflect the brain’s capacity to limit correlations that might become harmful to perceptual performance. We show that the state of short-time course noise correlations has a direct association with the animal’s performance, demonstrating a critical piece of evidence that the structure of correlations has a direct impact on behavioral decisions and sensory performance.

### Detecting and Interpreting Differential Correlations

Empirical evidence of differential correlations has rarely been reported, in part because of the prediction that they are so small (11) that thousands of neurons and thousands of trials are needed to detect them (12, 14). A major finding of this report is to present evidence (Figs. 9 and 10) that fluctuations in perceptual performance may reflect the impact of differential correlations on vision perception.

The magnitude of noise correlations is dynamic and changes with a number of factors (43), including state of consciousness (47), stimulus presentation (48), surround suppression (49), perceptual grouping (19, 50–52), attention (21, 23, 53, 54), and perceptual learning (20, 45). Our experiment brought many of these global factors under control by using highly trained, awake subjects that performed a demanding perceptual task.

Another advance we have made in detecting differential correlations is in how they are measured. Spike count correlation (*r*_SC_) is often used despite the fact that correlations vary over temporal scales (15–17, 37, 55). If differential correlations are rapid, then they could be masked by the slower correlations that are captured by *r*_SC_. By using the *r*_CCG_ measure introduced by Bair, Zohary, and Newsome (15), we found evidence of differential correlations operating at tens of milliseconds. This is much shorter than the width of the analysis windows that have typically been used to measure *r*_SC_ (43).

A complication is that the timescale of noise correlation is dynamic, so it can change within an area because of stimulation or behavioral state. This can be seen by comparing our results with previous studies of V1 (16, 37) and V4 (17) that used *r*_CCG_. They found noise correlations that saturated around 100 ms during stimulation, in both areas, but the subjects were either anesthetized (16, 37) or passively fixating (17).

We saw a similar *r*_CCG_ profile in the spontaneous responses of V1, but with visual stimulation presented under a task-relevant contingency, *r*_CCG_ saturated at 20 ms. In V4, saturation was also near 20 ms—before and during stimulation. Our results here are more closely compatible with other cases in which *r*_CCG_ also saturates at tens of milliseconds in the dorsal area V5/MT (middle temporal area) when monkeys perform a perceptual task (15, 52). Thus, the rapid, differential correlations that we observed here may only be readily detectable during the performance of a perceptual task.

### Recording Depth and Topography

We used 1-mm electrodes, with the result that our recordings were likely from the supragranular layers near the layer 3–4 border, from a mixture of neurons that projected nearby or to other cortical areas (56). One may predict that the measured correlations carry a stronger differential component if many of the recorded neurons project locally. This stems from the proposal that differential correlations build up between neurons that share afferent input from noisy sources (57). Thus a different approach to recording of neurons may yield samples with stronger or weaker differential correlations. Given the functional heterogeneity across layers in V1 and V4 (26, 55, 58, 59), cross-laminar recordings will be required to resolve this issue.

Some of the signals from V1 will have been relayed to V4 by other areas, but there are direct axon projections from V1 to V4 and from V4 to V1 (60). Although V1 and V4 neurons were coactivated with a single stimulus, the measured V1 RFs were not perfectly coincident with those of V4. Hence, some of the excitatory drive for the V4 neurons presumably came from other, unmeasured V1 neurons. Importantly, we saw qualitatively similar V4 correlations in a set of control experiments with the RDS centered on the aggregate V4 RF (not shown).

In V1 and V4, noise correlations span a range of cortical (i.e., retinotopic) distances when neurons respond to the same stimulus (16, 17, 51, 53)—which we observed [mean *r*_CCG_(20 ms); V1 pairs < 1.44 mm apart = 4.987e−2; V1 > 1.44 mm = 1.805e−2; V4 < 1.44 mm = 3.416e−2; V4 > 1.44 mm = 2.279e−2; all *t* test *P* → 0; median distance = 1.44 mm). Moreover, noise correlations in both areas increase when neurons respond to a common stimulus among an array of stimuli (21, 51). Thus, it is likely that the correlations of perceptually grouped neurons vary systematically across the cortex. We suggest that by stimulating V1 and V4 with the same object we increased the likelihood of the visual system having grouped them into a perceptual pool.

### Mechanisms of Noise Removal

In a sense, our results are puzzling. Neuronal correlations inside areas V1 and V4 show positive relationships between the neuron pairs with similar stimulus tuning and tuning curve slope (differential correlations). This kind of pattern is reported for motion in V5/MT (61) and orientation in V1 (19). With simple feedforward connections, one ought to observe the same relationship between V1 and V4. However, the general pattern of V1/V4 correlations is inverted (Fig. 3, *D*–*F*, Fig. 5, *E* and *F*, Fig. 6, *G–I*; Ref. 8).

Noise correlations like that can be removed linearly without losing information (8, 10). Thus, our simple model (Fig. 11) suggests that the combined action of V1 and V4 attenuates the local correlations through linear operations. However, differential correlations cannot be linearly removed by later processing (11, 18, 57). One way of resolving this discrepancy rests on our result that the covariance structure between areas is not static but evolves across temporal scales. Notably, the V1/V4 pattern emerged at slower timescales than the rapid differential correlations within each area.

Related results come from MT (50) and V4 (21), in which positive noise correlations strengthened between neurons with opposite tuning (but see Ref. 19). In V1, positive correlations that increase the information in linearly pooled responses can appear between neurons that respond to different stimuli (51). Finally, to change the V1 to V4 correlation structure in a trial would require the kind of fast and highly specific adaptations that are observed between them in attention tasks (62, 63).

Adaptable covariance may imply an underlying, nonlinear computation. But what could be biologically plausible? A spike count variance code could overcome correlations if correlated inputs were high-pass filtered in a spatial domain (e.g., across neurons) followed by a quadratic nonlinearity to compute variance (64). Another potential choice is divisive normalization (49, 65), which can model the structure of correlations between areas V1 and MT (22). Divisive normalization might decorrelate responses in two ways (see Eq. 3.1 in Ref. 66). First, spike count variance can be saturated by the correlated variance of other neurons, attenuating correlations in the normalized response. Second, the normalized responses of correlated units can move in opposite directions to their raw values, decorrelating them. One mechanism need not exclude others, and the brain might profit from the parallel action of several computations.

The challenge of removing differential correlations may ultimately depend upon where they come from. If they result from the external stimulus (57) or intrinsic thermodynamic sources of noise (67, 68), then they will be unknown to the brain. In this scenario, an adaptive mechanism is required to remove differential correlations. On the other hand, it is possible that differential correlations in sensory areas have a purely top-down origin (69, 70) that is known to the brain. In this case, removing differential correlations could be a trivial matter of subtracting away some kind of efference copy of the top-down signal that caused it (14, 19).

### Sources of Differential Correlations

The present analysis suggests both bottom-up (57) and top-down (69) origins of differential correlations. In the former, stimulus noise is sent through diverging projections to downstream areas; differential correlations then build up between neurons with common noisy inputs (57), especially if suboptimal computations are used (71). In the latter, noise in the selection of an attended feature value can lead to a gain profile that shifts position relative to the stimulus preferences of a population of neurons, inducing differential correlations (69).

Our results invite a compromise. Both sources are present, but they take effect at different moments. The initial burst of firing is a bottom-up event in which information is most reduced by rapid noise correlations, which are unlikely to have a top-down origin (55). In our data, only the Initial responses could reach the decorrelated information level, at a temporal scale of 100 ms. The Steady state information did increase at wider temporal scales yet failed to hit the decorrelated level within an 800-ms timescale. One interpretation is that rapid differential correlations in the Initial response had a bottom-up source, followed by slow differential correlations with a top-down source that could not be locally attenuated. This fits well with the common reports of top-down or contextual effects that follow the initial response of visual neurons to a stimulus (49, 53, 72–77).

This could explain why it was that only rapid correlations between V1 and V4 appeared to disrupt detection of the target RDS (Fig. 10*C*). If slow sources of differential correlation had a top-down origin that were known to the brain (e.g., Ref. 78), then these could plausibly be removed through simple subtraction. On the other hand, rapid, bottom-up differential correlations were not explicitly known and required a slower, adaptive mechanism to be removed. Under the conditions of our recording experiments, this mechanism did not have time to clean up the disparity signal that informed the subject’s choice.

### Neural Pooling and Perception

The statistical relationship between a pool of sensory neurons and perceptual behavior is connected to the noise correlations within the pool (7, 18, 79–81). That relationship may evolve over time, with a sensory pool driving a brief, bottom-up effect on behavioral choice, followed afterwards by a top-down reflection of choice bias (82). These effects are difficult to separate in many experiments, which employ long stimulus viewing times (18, 40, 72, 73, 83, 84).

However, a subject may base perceptual decisions on only a few hundred milliseconds of viewing in natural conditions (85). Hence, studies using mixtures of reaction time tasks and short-duration stimuli often observe that the transient response is accounted for by a small pool of neurons (25, 86–88) with a bottom-up link to behavior (89–91). Our results suggest that the transient response is a high-fidelity signal that could be decoded from a small pool of neurons. Later, top-down signals may arrive that impose slow and widespread differential correlations in a larger pool, thus injecting choice correlations into neurons that are never read out (18, 53, 72, 84, 92).

Although the associations between behavioral decisions and network state were stronger in one monkey than the other, the timing of these changes of state, in terms of differential correlations, supports this interpretation. Rapid fluctuations in the state of the network that mimic the change of state that occurs upon the presentation of the discrimination stimulus are detrimental to stimulus detection. This is a fundamental prediction of the theoretical role of differential correlations (9) that we have been able to validate with these experimental measures.

### Correlations and the Organizational Structure of the Neocortex

Our results lead us to propose a general framework for thinking about the effect of noise correlations on neuronal processing. First, within-area noise correlations that potentially limit sensory discrimination are a by-product of local information processing (56, 57). Second, we suggest that the signaling between areas acts to remove the harmful consequences of these local, intrinsic correlations. The removal process may use at least two strategies: one is the connectivity between cortical areas (e.g., Fig. 8), and the other is dynamic processing that adapts to the ongoing statistics of the neural signals. An implication of this framework is that one function of keeping diverse neocortical areas may be to contain the impact of differential correlations that arise from local neuronal processing within the circuitry of individual areas.

## GRANTS

This work was funded by MRC Grant MR/K014382/1 and Wellcome Trust Strategic Award 101092/Z/13/Z.

## DISCLOSURES

No conflicts of interest, financial or otherwise, are declared by the authors.

## AUTHOR CONTRIBUTIONS

A.J.P. conceived and designed research; J.E.T.S. performed experiments; J.E.T.S. analyzed data; J.E.T.S. and A.J.P. interpreted results of experiments; J.E.T.S. prepared figures; J.E.T.S. drafted manuscript; J.E.T.S. and A.J.P. edited and revised manuscript; J.E.T.S. and A.J.P. approved final version of manuscript.
